# DeepAR: a novel deep learning-based hybrid framework for the interpretable prediction of androgen receptor antagonists

**DOI:** 10.1186/s13321-023-00721-z

**Published:** 2023-05-06

**Authors:** Nalini Schaduangrat, Nuttapat Anuwongcharoen, Phasit Charoenkwan, Watshara Shoombuatong

**Affiliations:** 1 Center for Research Innovation and Biomedical Informatics, Faculty of Medical Technology, Mahidol University, Bangkok, 10700 Thailand; 2 Department of Community Medical Technology, Faculty of Medical Technology, Mahidol University, Bangkok, 10700 Thailand; 3 Modern Management and Information Technology, College of Arts, Media and Technology, Chiang Mai University, Chiang Mai, 50200 Thailand

**Keywords:** Androgen receptors, QSAR, Cheminformatics, Machine learning, Deep learning, Bioinformatics

## Abstract

Drug resistance represents a major obstacle to therapeutic innovations and is a prevalent feature in prostate cancer (PCa). Androgen receptors (ARs) are the hallmark therapeutic target for prostate cancer modulation and AR antagonists have achieved great success. However, rapid emergence of resistance contributing to PCa progression is the ultimate burden of their long-term usage. Hence, the discovery and development of AR antagonists with capability to combat the resistance, remains an avenue for further exploration. Therefore, this study proposes a novel deep learning (DL)-based hybrid framework, named DeepAR, to accurately and rapidly identify AR antagonists by using only the SMILES notation. Specifically, DeepAR is capable of extracting and learning the key information embedded in AR antagonists. Firstly, we established a benchmark dataset by collecting active and inactive compounds against AR from the ChEMBL database. Based on this dataset, we developed and optimized a collection of baseline models by using a comprehensive set of well-known molecular descriptors and machine learning algorithms. Then, these baseline models were utilized for creating probabilistic features. Finally, these probabilistic features were combined and used for the construction of a meta-model based on a one-dimensional convolutional neural network. Experimental results indicated that DeepAR is a more accurate and stable approach for identifying AR antagonists in terms of the independent test dataset, by achieving an accuracy of 0.911 and MCC of 0.823. In addition, our proposed framework is able to provide feature importance information by leveraging a popular computational approach, named SHapley Additive exPlanations (SHAP). In the meanwhile, the characterization and analysis of potential AR antagonist candidates were achieved through the SHAP waterfall plot and molecular docking. The analysis inferred that *N*-heterocyclic moieties, halogenated substituents, and a cyano functional group were significant determinants of potential AR antagonists. Lastly, we implemented an online web server by using DeepAR (at http://pmlabstack.pythonanywhere.com/DeepAR). We anticipate that DeepAR could be a useful computational tool for community-wide facilitation of AR candidates from a large number of uncharacterized compounds.

**Supplementary Information:**

The online version contains supplementary material available at 10.1186/s13321-023-00721-z.

## Introduction

Drug resistance represents a major obstacle to therapeutic innovations and are a prevalent feature in various cancers. The common incidence of resistance to therapeutic agents manifests through several mechanisms which allow for cancers to progress to its lethal stage [1]. One such cancer where resistance is common and often results in a severe occurrence is prostate cancer (PCa). PCa ranks as the fourth most commonly diagnosed cancer worldwide [2]. According to the International Agency for Research on Cancer, the estimated numbers of new PCa cases will rise from a total of 20,707,048 cases in 2020 to approximately 31,123,508 cases in 2040 with an increase of 60.5% seen in Asia [3]. Androgens are important for the regulation of prostate function by managing their proper growth and development [4–6]. Belonging to the nuclear receptor family, the androgen receptor (AR) shares genetic similarities with other well characterized receptors such as estrogen receptor (ER), progesterone receptor (PR), glucocorticoid receptor (GR) and mineralocorticoid receptor (MR) that are prominently involved in cancers such as breast, ovarian and prostate cancers, to name a few.

AR is comprised of the N-terminal domain, the DNA-binding domain and the Ligand binding domain (LBD) which make up its three main structural domains [7]. The ligand binding site or active site located in the LBD, is well characterized and engages with the ligand (i.e., agonist or antagonist) to modulate downstream action of AR [7, 8]. AR signaling allows for the survival and proliferation of PCa cells which are precariously dependent on androgen stimulation. Therefore, the hallmark target for therapeutic agents in PCa involves the inhibition of androgen synthesis by preventing the transcription of AR activity either through chemical castration (i.e., abiraterone acetate) [9] or AR antagonists (i.e., enzalutamide) [10, 11]. AR inhibitors such as enzalutamide, flutamide, bicalutamide and darolutamide which are in current clinical use, target the ligand binding pocket located in the LBD [12]. AR antagonists bind to the receptor by competing with endogenous androgens to block the transcription activity of AR [8]. Out of the two broad types of AR antagonists (i.e., steroidal and nonsteroidal), nonsteroidal compounds do not cross react with other steroid receptors (i.e., PR, MR, ER or GR) and show improved oral bioavailability. Hence, they are more compelling for clinical applications.

The survival rate of PCa patients have been vastly enhanced due to successful treatments with AR antagonists for androgen dependent PCa. On the other hand, consuming incessant AR antagonists leads to the rapid occurrence of resistance in the LBD active site [13]. These AR variants can contribute to PCa progression by transforming AR antagonists to agonists [14]. In addition, to date, the antagonist binding mode of AR has not been illuminated due to the absence of an AR-antagonist bound crystal structure. The ligand binding pocket of both AR antagonists and agonists are the same amino acids from helix 3, helix 4, helix 5, helix 11 and helix 12 forming polar and non-polar interactions. Among them, amino acids that form hotspots for the receptor specific binding through hydrogen bond interactions include Gln711 (H3), Met745 (H5), Arg752 (H5), Asn705 (H3) and Thr877 (H11) [8, 15–17]. Meanwhile, other auxillary surface-exposed ‘pockets’ such as the activation function-2 (AF2) site are also present in the LBD of AR. The AF2 site is essential for coactivator binding and encompasses a hydrophobic groove composed of numerous residues (such as Val716, Met734, Ile737, Gln738, and Met894) and flanked by charged residues (such as Gln733, Lys720). In 2007, the first crystal structure of AF2 in complex with antagonist was solved and since then, several other structures have been elucidated [18, 19]. An advantage of small molecule inhibition at the AF2 site is the direct disruption of coactivator recruitment as opposed to the traditional AR antagonists which act indirectly by inducing conformational change to prevent coactivator binding [18–21]. Therefore, pursuing the AF2 binding site could not only serve as a strategy to combat long-term AR-antagonists induced resistance but also offer an alternate pharmacological target.

Moreover, the process of drug discovery in its conventional form is expensive, time-consuming, and labor-intensive. Thus, the use of computer-aided drug discovery methodologies (i.e., molecular docking, molecular dynamic (MD) simulations, quantitative structure–activity relationship (QSAR), and deep learning (DL)), have been frequently employed to alleviate such burdens over the past two decades. Such studies utilized machine learning (ML)-based, structure-based and ligand-based approaches to discover potential AR modulators [22–29]. ML-based approaches have made significant strides in constructing QSAR models that can handle large biological datasets while maintaining interpretability [30]. DL-based techniques have also advanced significantly in recent years and are proving to be useful in drug modeling due to the growing availability of biological data. For instance, Elmarakeby et al. [31] developed a biologically informed DL model, which were capable of stratifying patients with prostate cancer by their treatment-resistance state and identifying molecular drivers of resistance for targeted therapy. Cherkasov et al. [29] employed deep neural networks to create DL-based models to predict the response of resistant mutations to anti-androgens and testosterone. Idakwo et al. [32] compared DL-based and random forest (RF)-based models for predicting AR chemical toxicity and found that the DL-based models outperformed RF-based models by over 20% with statistical significance. Yu et al. [33] utilized 2-D chemical structure image information as input for creating their DL-based model in order to predict agonist activity for AR toxicity. However, despite the progress made in these studies, there is a lack of interpretable DL-based approaches for predicting AR antagonists that can be deployed as a web server for community-wide usage.

**Fig. 1:**
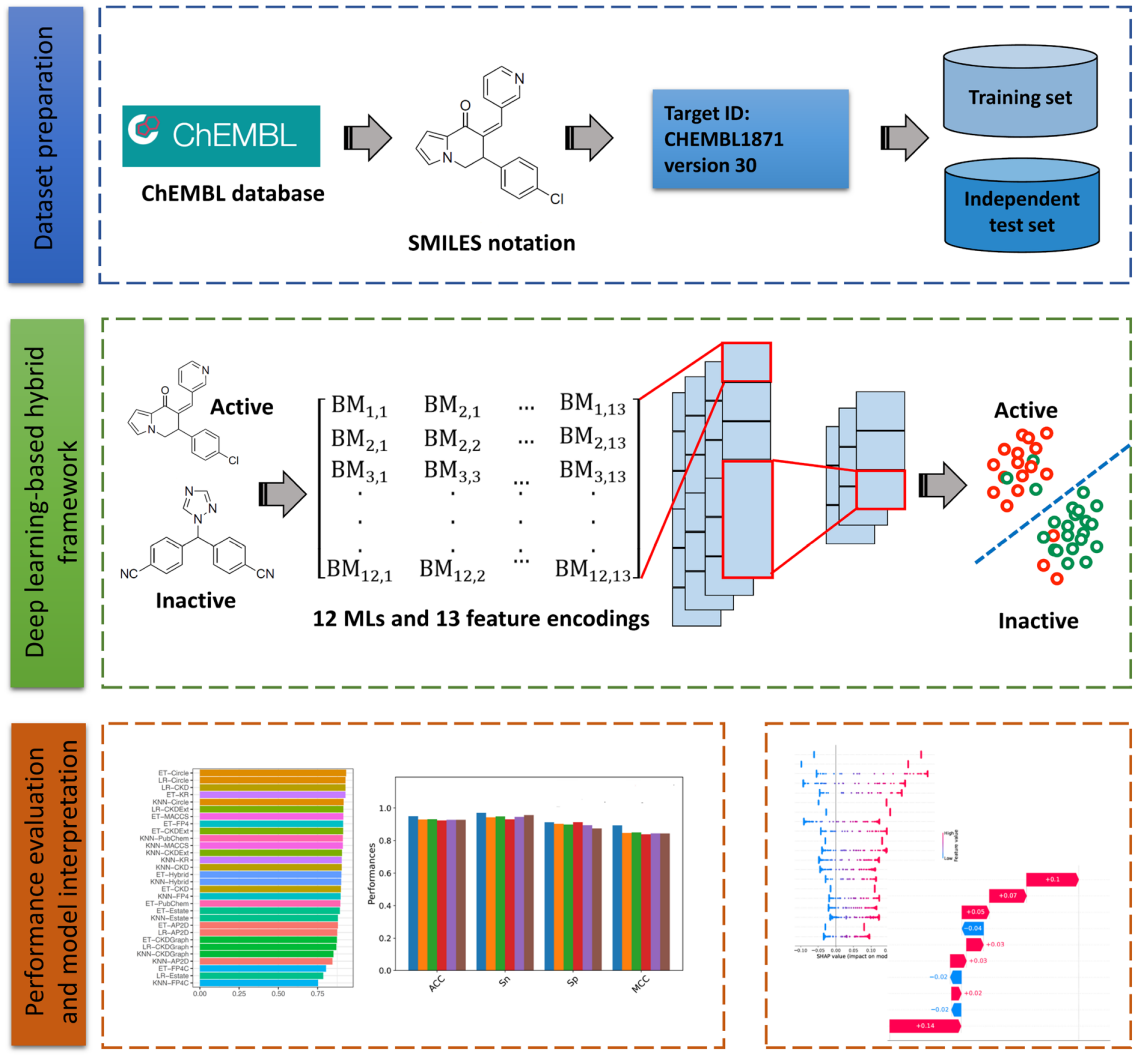
System flowchart of the proposed DeepAR. The overall workflow for the development of DeepAR contains three major steps: dataset preparation, DeepAR optimization and construction, performance evaluation and model interpretation

Keeping these limitations in mind, we develop DeepAR, a DL-based hybrid framework for accurately and rapidly identifying AR antagonists. DeepAR is a structure-independent protocol, which is able to identify AR antagonists by using the SMILES notation without the use of structural information. The design and development process of DeepAR is summarized in Fig. 1. First, we established a benchmark dataset by collecting antagonists of AR from the ChEMBL database. Second, DeepAR employed 12 types of molecular descriptors and 13 different ML algorithms to construct 156 baseline models. Subsequently, these baseline models were utilized for generating 156 probabilistic features (PFs). Finally, a meta-model based on a one-dimensional (1D) convolutional neural network (1D-CNN) was developed by using the combination of all the 156 PFs and the stacking strategy. Both tenfold cross-validation and independent test results demonstrate that DeepAR outperformed several conventional ML classifiers. In addition, our proposed framework is able to provide the feature importance information by leveraging a popular computational approach, named SHapley Additive exPlanations (SHAP). Furthermore, the SHAP waterfall plot coupled with molecular docking was employed for the characterization and analysis of novel AR antagonists.

## Materials and methods

### Construction of training and independent test datasets

The training and independent test datasets were collected from the ChEMBL database (Target ID: CHEMBL1871; version 30) [34]. At first, 10,285 compounds exhibiting activity towards AR was downloaded and subjected to data curation using our in-house code, where compounds having the symbol ‘ = ’ in their “Standard Value” column were retained while those having symbols of ‘ < , > ,/’were removed. In addition, redundant and missing data were also discarded. After which, compounds with bioactivity unit of IC_50_ (half-maximal inhibitory concentration) with standard deviation of 2, were selected to form the final dataset which consisted of 1,309 compounds. The IC_50_ of a compound is a measure of the concentration needed to inhibit a specific biological activity by 50%. It serves as a widely accepted indicator of a compound's potency in drug discovery, with compounds possessing lower IC_50_ values generally considered to be more potent and exhibiting higher biological activity. To be specific, compounds with IC_50_ ≤ 1 μM were considered as active (positive samples), while compounds with IC_50_ ≥ 10 μM were considered as inactive (negative samples). As a result, the final dataset consisted of 433 active and 409 inactive compounds, respectively. Among these compounds, 346 active and 327 inactive compounds were randomly selected for the construction of the training dataset (named AR-TRN), while the remaining compounds were used to create the independent test dataset consisting of 87 active and 82 inactive compounds (named AR-IND).

### Chemical space analysis

Chemical space analysis is a valuable way for exploring, comprehending, and optimizing the vast range of potential compounds, and identifying promising new drug candidates. As mentioned above, all molecules were categorized into active and inactive groups based on their IC50 values. Here, we computed, graphed, and compared eight physicochemical properties related to Lipinski's Rule of Five (Ro5) and molecular complexity for both groups, including molecular weight (MW), the Ghose-Crippen-Viswanadhan octanol-water partition coefficient (ALogP), number of hydrogen-bond acceptors (nHAcc), number of hydrogen-bond donors (nHDon), aromatic ratio (ARR), number of rings (nCIC), number of rotatable bonds (RBN), and number of benzene-like rings (nBnz). The Dragon software (version 6) [35] was employed to compute the molecular complexity descriptors, whereas in-house scripts using ggplot2 package [36] in R program (version 4.2.2 [37]) were utilized to graph Lipinski's Ro5 descriptors. In addition, we performed maximal, minimal, median, and mean values as well as determined statistical significance using *p*-values based on the Mann-Whitney U test (at the level of *p* < 0.001).

### Molecular descriptor engineering

QSAR studies utilize molecular fingerprints to gather data with reference to substructures natively present in molecules or compounds of interest. In this study, we used the PADEL-descriptor software [38] to remove salt and normalize tautomers as part of the pre-processing step for standardizing compounds. The structural features of the investigated compounds were obtained by using the SMILES notation as input values for the calculation of 12 molecular fingerprint descriptors (i.e., CKD, CKDExt, CKDGraph, AP2D, KR, MACCS, Circle, Estate, Hybrid, PubChem, FP4C and FP4). Table 1 highlights the details of each fingerprint descriptor. Herein, the Python environment was used for all molecular descriptor extractions [39].

**Table 1:** Summary of twelve molecular fingerprints used in this study

Fingerprint	Abbreviation	#Feature	Description	Ref.
2D atom pair	AP2D	780	Presence of atom pairs at various topological distances	[83]
CDK	CKD	1024	Fingerprint of length 1,024 and search depth of 8	[84]
CDK extended	CKDExt	1024	Extends the fingerprint with additional bits describing ring features	[84]
CDK graph only	CKDGraph	1024	A special version that considers only the connectivity and not bond order	[84]
Circle	Circle	1024	Circular fingerprint	[85]
EState	EState	79	Electrotopological state atom types	[86]
Hybrid	Hybrid	1024	CDK hybridization fingerprint	[85]
Klekota–Roth	KR	4860	Presence of chemical substructures	[87]
MACCS	MACCS	166	Binary representation of chemical features defined by MACCS keys	[88]
Pubchem	Pubchem	881	Binary representation of substructures defined by PubChem	[89]
Substructure	FP4	307	Presence of SMARTS patterns for functional groups	[90]
Substructure count	FP4C	307	Count of SMARTS patterns for functional groups	[90]

### Deep learning-based hybrid framework of DeepAR

Herein, DeepAR was developed based on the stacking learning strategy. This strategy has been shown to provide an outstanding performance compared with single-feature-based models [40–44]. Specifically, the construction of the proposed DeepAR involves two main phases: baseline and meta models’ development (as illustrated in Fig. 1). In the first phase, we employ different ML algorithms and feature encodings to develop baseline models. The output of the first phase is used as the input to develop the meta-model based on a DL algorithm, in the second phase.

#### The first phase

In this phase, we applied 12 well-known feature encodings to extract samples in the AR-TRN dataset, including CKD, CKDExt, CKDGraph, AP2D, KR, MACCS, Circle, Estate, Hybrid, PubChem, FP4C, and FP4. These molecular descriptors are widely used to represent several types of inhibitors [41, 45–48]. In the meanwhile, 13 popular ML algorithms were selected for the construction of baseline models, including RF, AdaBoost (ADA), light gradient boosting machine (LGBM), partial least squares (PLS), multilayer perceptron (MLP), naive Bayes (NB), decision tree (DT), extremely randomized trees (ET), extreme gradient boosting (XGB), k-nearest neighbor (KNN), logistic regression (LR), support vector machine (SVM) combined with linear (SVMLN) and radial basis function (SVMRBF) kernels. As a result, we obtained a total of 156 baselines, which were trained and optimized using the scikit-learn package (Additional file 1: Table S1). In addition, we comprehensively investigated the contribution of the 12 feature encodings and 13 ML algorithms in AR antagonist prediction based on the tenfold cross-validation and independent tests. Herein, we determine the best-performing model in terms of cross-validation MCC.

#### The second phase

After obtaining 156 baselines, we utilized them to generate a feature vector for the construction of the meta-model. For a given compound *C*, each baseline model can provide the PF, which is in the range of 0–1. The feature vector ($\mathrm{FV}(C)$) based on the 156 baselines can be defined by

1 $${\text{FV}}\left( C \right) = \left\{ {PF_{{{\text{BM}}_{1,1} }} , PF_{{{\text{BM}}_{1,1} }} ,PF_{{{\text{BM}}_{1,1} }} , \ldots ,PF_{{{\text{BM}}_{{{\text{i}},{\text{j}}}} }} , \ldots PF_{{{\text{BM}}_{13,12} }} } \right\}$$

where ${PF}_{{\mathrm{BM}}_{\mathrm{i},\mathrm{j}}}$ is the PF derived from the baseline model trained with the *i*^*th*^ ML algorithm in conjunction with the *j*^*th*^ feature encoding. As a result, $\mathrm{FV}(C)$ was converted into a 156-dimensional (D) feature vector. In this study, we applied 1D-CNN for the construction of the meta-model (named mCNN) because of its built-in capability of feature design and extraction [49–54]. For the mCNN, it was developed by using a single convolutional layer containing three region sizes (i.e., 3, 4, and 5) and each of region sizes involved 100 filters [55]. As a result, we obtained a total of 300 filters to perform 1-D convolution on the 156-D feature vector and created six feature maps. After that, the six feature maps were used to generate a 6-D feature vector. Finally, the 6-D feature vector was used as input in the softmax layer for the prediction of compound *C* to be active or inactive against AR. In order to maximize the utilization of mCNN, we utilized the grid search approach to determine its optimal parameters (epochs $\in$ {20, 50, 100, 200} and learning rate $\in$ {0.00001, 0.0001, 0.001, 0.01) by performing tenfold cross-validation on the AR-TRN dataset.

### Evaluation criteria

To assess the predictive capability of our proposed model, we employed six well-known metrics, including F1, sensitivity (Sn), specificity (Sp), Matthew’s coefficient correlation (MCC), accuracy (ACC), and the area under the receiver operating characteristics (ROC) curve (AUC). These metrics are described as follows [40, 56, 57]:

2 $${\text{F}}1 = 2 \times \frac{{{\text{TP}}}}{{2{\text{TP}} + {\text{FP}} + {\text{FN}}}}$$

3 $$Sn = \frac{TP}{{\left( {TP + FN} \right)}}$$

4 $$Sp = \frac{TN}{{\left( {TN + FP} \right)}}$$

5 $$MCC = \frac{TP \times TN - FP \times FN}{{\sqrt {\left( {TP + FP} \right)\left( {TP + FN} \right)} \,\left( {TN + FP} \right)\left( {TN + FN} \right)}}$$

6 $$ACC = \frac{TP + TN}{{\left( {TP + TN + FP + FN} \right)}}$$

where TP, FP, TN, and FN the number of true positive, false positive, true negative, false positive and false negative compounds, respectively.

### Molecular docking

Herein, we collected a set of 3,811 compounds described with various cell-based assays (i.e., EC_50_, K_i_, K_d_, potency and percentage inhibition) from the CHEMBL database [34]. Among these compounds, DeepAR was employed to identify which compound was the most potential one, where the compounds having the highest probability scores were deemed as promising compounds having activity against AR. Please note that these compounds were not found in the AR-TRN and AR-IND datasets. Molecular docking was performed to investigate the binding modality of the ten top-ranked compounds (PDB ID: 2YHD) [19]. The protein structures were prepared by extracting the co-crystal ligand, removing water molecules and calculating the Gasteiger charges using MGLTools [58]. OpenBabel was used to optimize and generate structures with low-energy conformers of the input ligands [59]. The grid boxes were generated and a seed number of 1000 was defined using the default paraments of the Autodock Vina software [60]. Upon redocking, the calculated RMSD between the co-crystal ligand and its re-docked ligand was 2.32 Å, which is satisfactory for further investigation. Consequently, the built-in scoring function was utilized for calculating the binding energy of the predicted AR antagonists. The binding modality of all docked ligands were analyzed using Protein–ligand interaction Profiler (PLIP) [61] and visualized using PyMOL (Schrodinger, Inc.).

## Results and discussion

### Exploratory data analysis

In this section, we performed the chemical space analysis to characterize the patterns between active and inactive compounds. Initially, the general chemical space was visualized based on MW versus ALogP. Additionally, the Ro5 descriptors were employed to compare the active and inactive compounds. Ro5 determines the drug likeness of compounds based on their molecular properties including MW (< 500), ALogP (< 5), nHAcc (< 10), and nHDon (< 5) [62]. The visualization of the MW chemical space as a function of ALogP is displayed in Additional file 1: Fig. S1. The majority of compounds were clustered within the MW range of 200–500 Da with an ALogP between 1 and 6. Furthermore, Additional file 1: Fig. S2 shows the distribution of active and inactive compounds based on the Ro5 descriptors. It was observed that the compounds adhered to the Ro5 criteria with a MW of less than 500 Da, ALogP less than 5, and nHBDon and nHBAcc less than 10. The statistical analysis computed through the Mann–Whitney *U* test revealed a significant difference (*p* < 0.001) between the active and inactive compounds in terms of MW. Most of the active compounds had lower MW (347.15 ± 85.69) than inactive compounds (364.13 ± 87.57), as observed from the mean values of boxplots in Additional file 1: Fig. S2. Similarly, nHBAcc values of 3.32 ± 2.07 and 3.62 ± 2.01 were significantly different between the active and inactive compounds, respectively. However, the ALogP values for active (3.71 ± 1.14) and inactive (3.92 ± 1.38) compounds were only slightly significant. Additionally, both active and inactive compounds had similar nHBDon values, which were not statistically significant.

Furthermore, the clinical success of a compound depends on various factors, including its molecular complexity, which is determined by properties such as aromaticity, the number of rings, chiral centers, fused rings, functional groups, and the number of rotatable bonds [54]. These properties, in turn, can impact crucial biological events such as solubility, oral bioavailability, and toxicity [55]. In this study, we analyzed four descriptors –ARR, nCIC, RBN, and nBnz—to determine the molecular complexity of the studied compounds and compared them between the active and inactive groups. Additional file 1: Fig. S3 displays a box plot of these descriptors. Our results indicate that active compounds possess a lower ARR ratio, fewer rotatable bonds, and benzene-like rings compared to inactive compounds, and these differences are statistically significant (*p* < 0.001).

### Overall prediction results from different ML algorithms and molecular descriptors

**Fig. 2:**
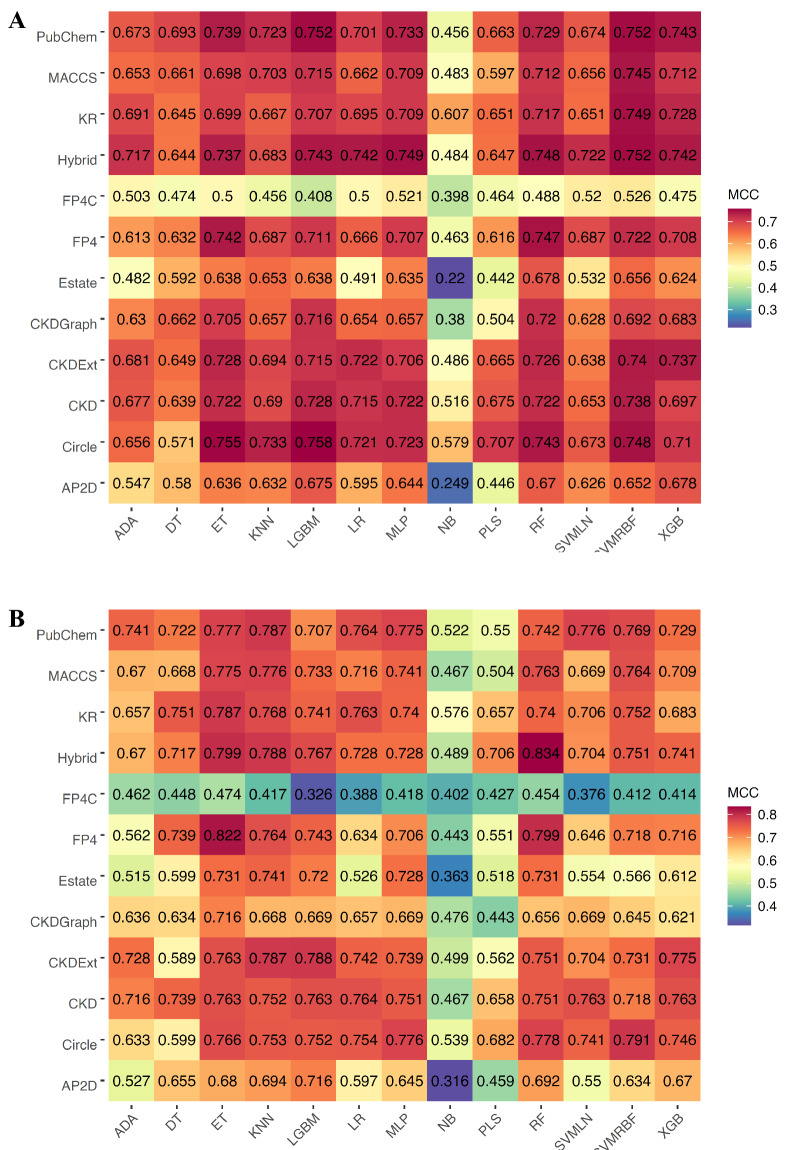
MCC values of 156 baseline models in terms of tenfold cross-validation (**A**) and independent (**B**) tests

In this section, we conducted a comparative analysis of 156 ML classifiers trained with 13 ML algorithms and 12 molecular descriptors. The performance of each classifier was evaluated based on both tenfold cross-validation and independent tests. As mentioned above, the ML classifier having the highest cross-validation MCC was deemed as the best-performing model. Figure 2 and Additional file 1: Tables S2–S4 show the performance of all the ML classifiers developed herein. We notice that the top five powerful ML classifiers consisted of LGBM-Circle, ET-Circle, SVMRBF-PubChem, LGBM-PubChem, and SVMRBF-Hybrid with respective MCC of 0.758, 0.755, 0.752, 0.752, and 0.752. In the meanwhile, Additional file 1: Table S4 shows that the top three important descriptors were Hybrid, Circle, and PubChem with respective average MCC of 0.701, 0.698, and 0.695. Interestingly, all of the top five ML classifiers were developed from these important descriptors. This indicates that Hybrid, Circle, and PubChem could be more important for AR antagonist prediction as compared with the remaining molecular descriptors. Based on the cross-validation results, LGBM-Circle was indicated as the best-performing model, while this model had MCC of 0.752 with ACC of 0.876 and AUC of 0.938 in terms of the independent test. On the other hand, RF-Hybrid provided the highest MCC of 0.834 with ACC of 0.917 and AUC of 0.935 in terms of the independent test. This evidence indicates that single-feature-based models could not provide a stable performance on both the AR-TRN and AR-IND datasets.

### Performance evaluation of DeepAR

In order to improve the stable performance of AR antagonist prediction, we integrated several ML classifiers to develop a meta-model by using the stacking strategy. Specifically, we employed CNN model in conjunction with the 156-D feature vector for the construction of the meta-model (DeepAR). As seen in Tables 2, 3, DeepAR achieves MCC of 0.762 and 0.823 in terms of the AR-TRN and AR-IND datasets, respectively. Remarkably, DeepAR provided ACC of 0.911, Sn of 0.897, Sp of 0.927, and AUC of 0.945 on the AR-IND dataset. In addition, we compared its performance with that of other meta-models trained with 13 ML algorithms and the same 156-D feature vector. In Table 2, we notice that DeepAR, mRF, and mLGBM achieved a similar performance and outperformed other meta-models in terms of cross-validation MCC with a range of 0.762–0.770. In case of the independent test results, ACC and MCC of DeepAR were 2.34 and 4.63–4.80% higher than mRF and mLGBM (Table 3).

**Table 2:** Cross-validation results of different stacked models on the training dataset

Meta-model	ACC	Sn	Sp	MCC	AUC	F1
DeepAR	0.880	0.861	0.899	0.762	0.941	0.880
RF	0.884	0.887	0.881	0.770	0.952	0.887
LGBM	0.880	0.898	0.859	0.762	0.945	0.885
SVMRBF	0.878	0.893	0.862	0.758	0.923	0.882
XGB	0.877	0.901	0.850	0.755	0.942	0.882
LR	0.877	0.901	0.850	0.755	0.946	0.883
PLS	0.877	0.893	0.859	0.755	0.946	0.881
NB	0.877	0.887	0.866	0.755	0.919	0.881
ET	0.875	0.887	0.862	0.753	0.949	0.880
MLP	0.874	0.881	0.866	0.750	0.938	0.877
KNN	0.869	0.887	0.850	0.739	0.869	0.874
SVMLN	0.866	0.887	0.844	0.735	0.921	0.872
ADA	0.854	0.867	0.841	0.711	0.923	0.860
DT	0.853	0.846	0.859	0.709	0.853	0.854

**Table 3:** Independent test results of different stacked models on the independent test dataset

Meta-model	ACC	Sn	Sp	MCC	AUC	F1
DeepAR	0.911	0.897	0.927	0.823	0.945	0.912
RF	0.888	0.862	0.915	0.777	0.941	0.888
LGBM	0.888	0.885	0.890	0.775	0.947	0.890
SVMRBF	0.864	0.851	0.878	0.728	0.913	0.865
XGB	0.864	0.839	0.890	0.729	0.949	0.864
LR	0.893	0.874	0.915	0.788	0.949	0.894
PLS	0.893	0.874	0.915	0.788	0.951	0.894
NB	0.888	0.862	0.915	0.777	0.906	0.888
ET	0.899	0.897	0.902	0.799	0.952	0.902
MLP	0.876	0.839	0.915	0.754	0.927	0.874
KNN	0.834	0.816	0.854	0.670	0.835	0.835
SVMLN	0.846	0.816	0.878	0.694	0.913	0.845
ADA	0.858	0.828	0.890	0.718	0.922	0.857
DT	0.822	0.816	0.829	0.645	0.823	0.826

### DeepAR is able to improve the predictive performance

**Fig. 3:**
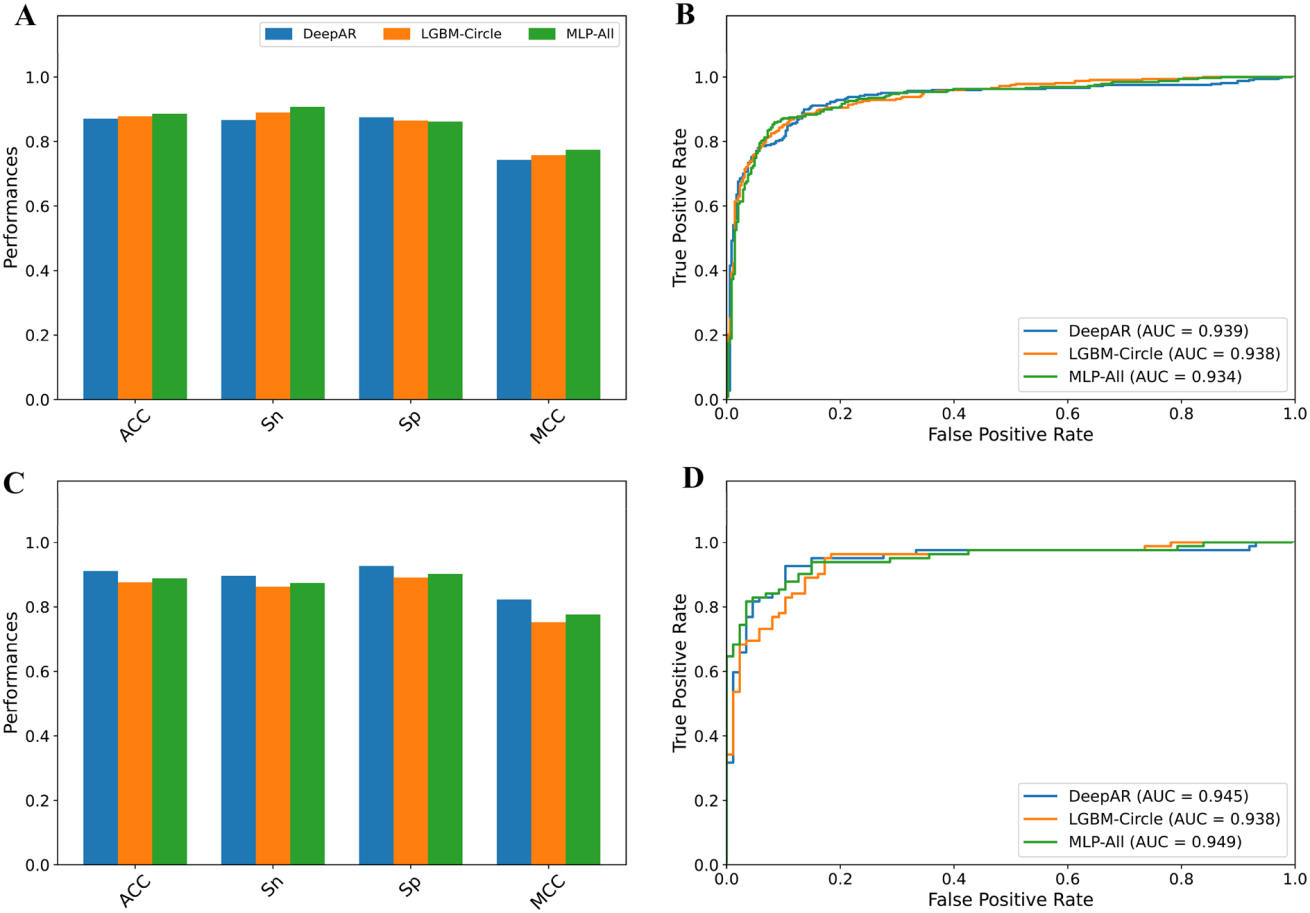
Performance comparison of DeepAR with LGBM-Circle and MLP-All on the Main-TRN (**A**, **B**) and Main-IND (**C**, **D**) datasets. Prediction results of StackPR with the top five baseline models in terms of MCC, Sn, Sp and MCC (**A**, **C**). ROC curves and AUC values of StackPR with the top five baseline models (**B**, **D**)

To show that our proposed DeepAR is better than other conventional ML classifiers, we designed two sets of the comparative analysis. For the first comparative analysis, we compared the performance of DeepAR with single-feature-based models. As can be seen from Table 4, DeepAR achieved an overall best performance compared with the best single-feature-based model (i.e., LGBM-Circle) in terms of ACC, Sp, MCC and AUC on both the AR-TRN and AR-IND datasets. On the AR-IND dataset, MCC, ACC and Sp of DeepAR were 7.10, 3.53, and 3.66% higher than the LGBM-Circle, respectively. In addition, we also compared the performance of DeepAR with ML classifiers trained with all the 12 molecular descriptors in the second comparative analysis. Additional file 1: Tables S5, S6 show that the highest MCC in terms of the tenfold cross-validation test is achieved by MLP (referred MLP-All herein). By comparing with MLP-All on the AR-IND dataset, DeepAR exhibited better MCC, ACC, Sn, and Sp with respective increase of 4.74, 2.34, 2.30, and 2.44% (Fig. 3 and Table 4). Taken together, these results confirmed the predictive capability of DeepAR for enhancing the AR prediction performance. Furthermore, its high Sp and MCC values reveal that the proposed DeepAR could precisely identify active AR compounds from a huge number of compounds found in several public databases.

**Table 4:** Performance comparison of DeepAR and conventional ML classifiers on the training and independent test datasets

Evaluation strategy	Method	ACC	Sn	Sp	MCC	AUC	F1
Cross-validation	LGBM-Circle	0.878	0.890	0.865	0.758	0.938	0.882
	MLP-All	0.886	0.907	0.862	0.774	0.934	0.891
	DeepAR	0.880	0.861	0.899	0.762	0.941	0.880
Independent test	LGBM-Circle	0.876	0.862	0.890	0.752	0.938	0.877
	MLP-All	0.888	0.874	0.902	0.776	0.949	0.889
	DeepAR	0.911	0.897	0.927	0.823	0.945	0.912

### Application of DeepAR to characterize AR antagonists

**Fig. 4:**
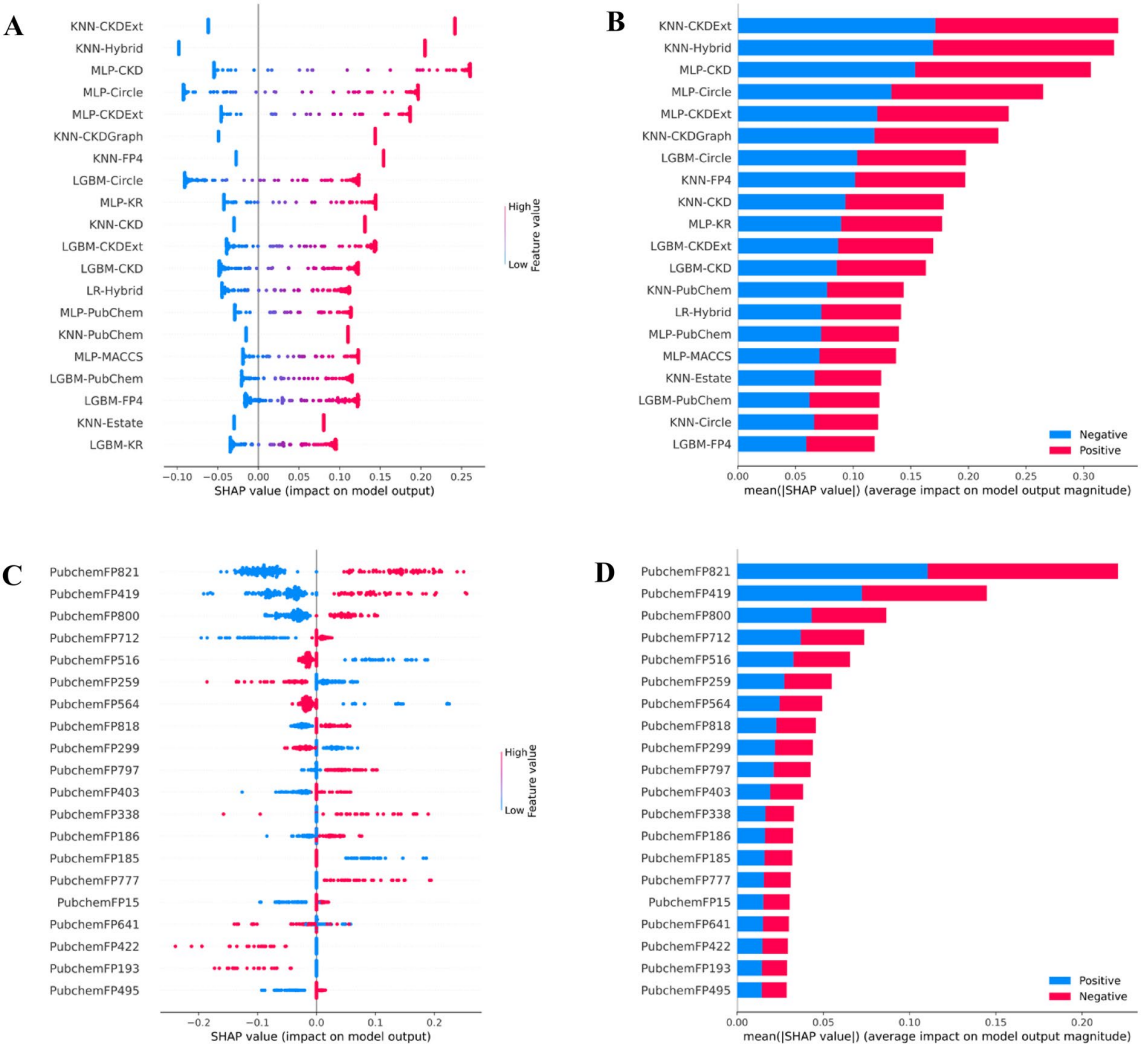
Feature importance from DeepAR (**A**, **B**) and LGBM-PubChem (**C**, **D**) as ranked by SHAP values based on the training dataset. **A**, **C** Magnitude and direction of the contribution of each feature to the model prediction of AR antagonists. **B**, **D** Mean absolute SHAP values, where positive and negatives SHAP values influences the predictions toward positive and negative samples, respectively

Herein, the popular SHAP framework [70] implemented by Lundberg and Lee [63] was utilized to reveal which features are beneficial for DeepAR. Specifically, features contributing to the global impact of active and inactive compounds are indicated by positive and negative SHAP values, respectively, where positive and negative SHAP values indicated on the positive and negative scales are highly impactful for active and inactive compound substructures, respectively. Figure 4A, B show that five top-ranked important features for DeepAR consist of KNN-CKDExt, KNN-Hybrid, MLP-CKD, MLP-Circle, and MLP-CKDExt. It is worth noting that the LGBM-PubChem model ranked at number 11. This model is considered interpretable due to its utilization of PubChem substructure fingerprint descriptors, which are known for their interpretability. Thus, we employed this model in conjunction with the SHAP framework to provide better understanding of potential substructures of AR antagonists. As seen in Fig. 4C, D along with Table 5, six out of the top-twenty informative features involve four nitrogen-containing (i.e., PubChemFP821, PubChemFP419, PubChemFP800, and PubChemFP338) and two aromatic features (i.e., PubChemFP797 and PubChemFP777). This indicates that compounds with nitrogen and aromatic features represent substructures having a high influence on AR antagonism. Exploring further into the description of the PubChem features (Table 5), provides insight that the nitrogen-containing features pertain to *N*-methylcyclohexanamine and a cyano group. These scaffolds are observed as part of an active substructure in extensively studied AR antagonists bicalutamide, apalutamide, enzalutamide, and darolutamide where the cyano group of the benzonitrile moiety has been identified as a key interaction involved in amino acid binding in the LBD [8]. In addition, nitrogen-containing heterocyclic moieties make up 75% of current market available drugs approved by the FDA as they exhibit anticancer pharmacological profiles [8, 64, 65].

**Table 5:** Summary of the top-twenty important features ranked by SHAP values along with their corresponding SMARTS patterns and substructure description

Feature	SMARTS pattern	Substructure description
PubChemFP821	CC1C(N)CCCC1	2-methylcyclohexan-1-amine
PubChemFP419	C≡N	Cyano group
PubChemFP800	CC1CC(N)CCC1	3-methylcyclohexan-1-amine
PubChemFP712	C–C(C)-C(C)-C	2,3-dimethylbutane
PubChemFP516	[#1]-C = C-[#1]	Ethene
PubChemFP259	≥ 3 aromatic rings	Greater than 3 cyclic rings
PubChemFP564	C = C–C = C	Buta-1,3-diene
PubChemFP818	CC1C(C)CCCC1	1,2-dimethylcyclohexane
PubChemFP299	N–H	Imidogen
PubChemFP797	CC1CC(C)CCC1	1,3-dimethylcyclohexane
PubChemFP403	N(:C)(:C)(:C)	*N,N*-dimethylmethanamine
PubChemFP338	C(~ c)(~ c)(~ H)(~ N)	Propan-2-amine
PubChemFP186	≥ 2 saturated or aromatic carbon-only ring size 6	Greater than 2 saturated or aromatic carbon-only six-member cyclic ring
PubChemFP185	≥ 2 any ring size 6	Greater than 2 six-member cyclic ring
PubChemFP777	cc1ccc(o)cc1	4-methylphenol
PubChemFP15	≥ 2 N	Greater than 2 nitrogen atoms
PubChemFP641	O-C–C-C = C	But-3-en-1-ol
PubChemFP422	N = N	Diazene
PubChemFP193	≥ 3 saturated or aromatic carbon-only ring size 6	Greater than 3 saturated or aromatic carbon-only six-member cyclic ring
PubChemFP495	C-N–C:C	*N*-methylethanamine

### Application of DeepAR for the large-scale identification of novel AR antagonists

In this section, we employed DeepAR to calculate the probabilities of 3,811 compounds in order to determine the most potential compounds having activity against AR. Table 6 lists the top-ten compounds with the highest probability scores of being AR antagonists, while Additional file 1: Fig. S4 shows the structures of the compounds. In addition, we conducted molecular docking to discern the binding mode and binding affinities of the compounds. As previously mentioned, the AF2 site offers an alternate binding target and thus, the crystal structure of AR with the antagonist bound to the allosteric AF2 site (PDBID: 2YHD) was used for the docking study. Table 6 shows that CHEMBL3233070, CHEMBL3238281, and CHEMBL3233072 achieved similar docking scores of − 6.8, − 6.9, and − 6.7 kcal/mol, respectively. To be specific, the ranks (probability, docking score) of the top-three compounds, CHEMBL3233070, CHEMBL3238281, and CHEMBL3233072 were (5, 2), (9, 1), and (10, 3), respectively. Thus, these three compounds were chosen for further investigation.

**Table 6:** Summary of the top ten compounds from DeepAR screening with their SMILES notation, probability and corresponding docking scores

CHEMBL ID	SMILES	Probability	Docking score (Kcal/mol)
CHEMBL3238279	C[C@](O)(COc1ccccc1Cl)C(= O)N1CCc2c(C#N)cccc21	0.96041	− 6.0
CHEMBL3233069	COc1ccc(OC[C@](C)(O)C(= O)N2CCc3c(C#N)cccc32)c(Cl)c1	0.96038	− 5.2
CHEMBL3238280	C[C@](O)(COc1ccc(Br)cc1)C(= O)N1CCc2c(C#N)cccc21	0.96033	− 6.5
CHEMBL3238276	C[C@](O)(COc1ccccc1F)C(= O)N1CCc2c(C#N)cccc21	0.96032	− 6.6
CHEMBL3233070	C[C@](O)(COc1ccc(Cl)cc1F)C(= O)N1CCc2c(C#N)cccc21	0.96030	− 6.8
CHEMBL3238274	C[C@](O)(COc1ccc(F)cc1)C(= O)N1CCc2c(C#N)cccc21	0.96029	− 6.4
CHEMBL3238278	C[C@](O)(COc1cccc(Cl)c1)C(= O)N1CCc2c(C#N)cccc21	0.96023	− 6.3
CHEMBL3238277	C[C@](O)(COc1ccc(Cl)cc1)C(= O)N1CCc2c(C#N)cccc21	0.96022	− 6.4
CHEMBL3238281	C[C@](O)(COc1ccc(C(F)(F)F)cc1)C(= O)N1CCc2c(C#N)cccc21	0.96022	− 6.9
CHEMBL3233072	C[C@](O)(COc1ccc(Br)cc1F)C(= O)N1CCc2c(C#N)cccc21	0.96022	− 6.7

**Fig. 5:**
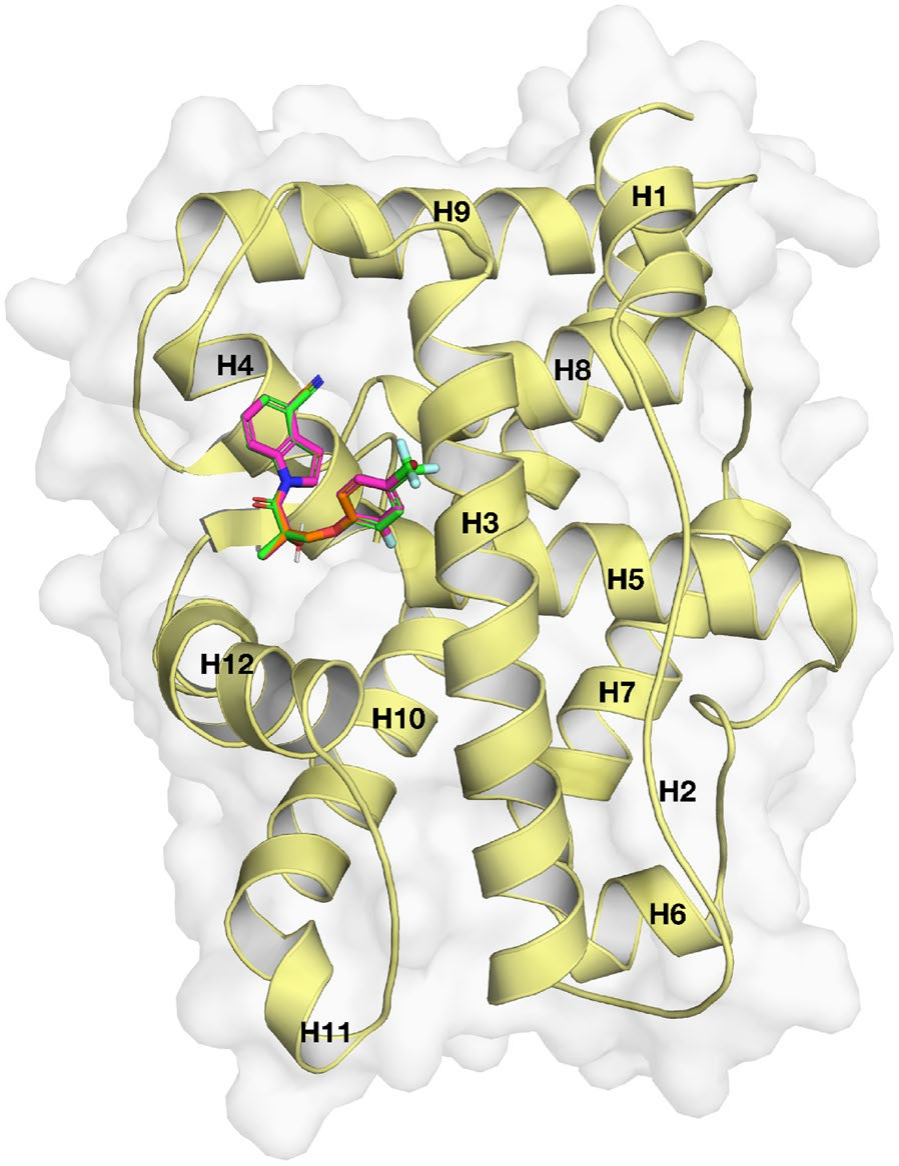
Superimposed docked pose of AR (PDB ID: 2HYD) and the top three compounds with highest probabilities as measured by DeepAR where green, orange and magenta colours represent the carbon backbone of CHEMBL3238281, CHEMBL3233070 and CHEMBL3233072, respectively

**Fig. 6:**
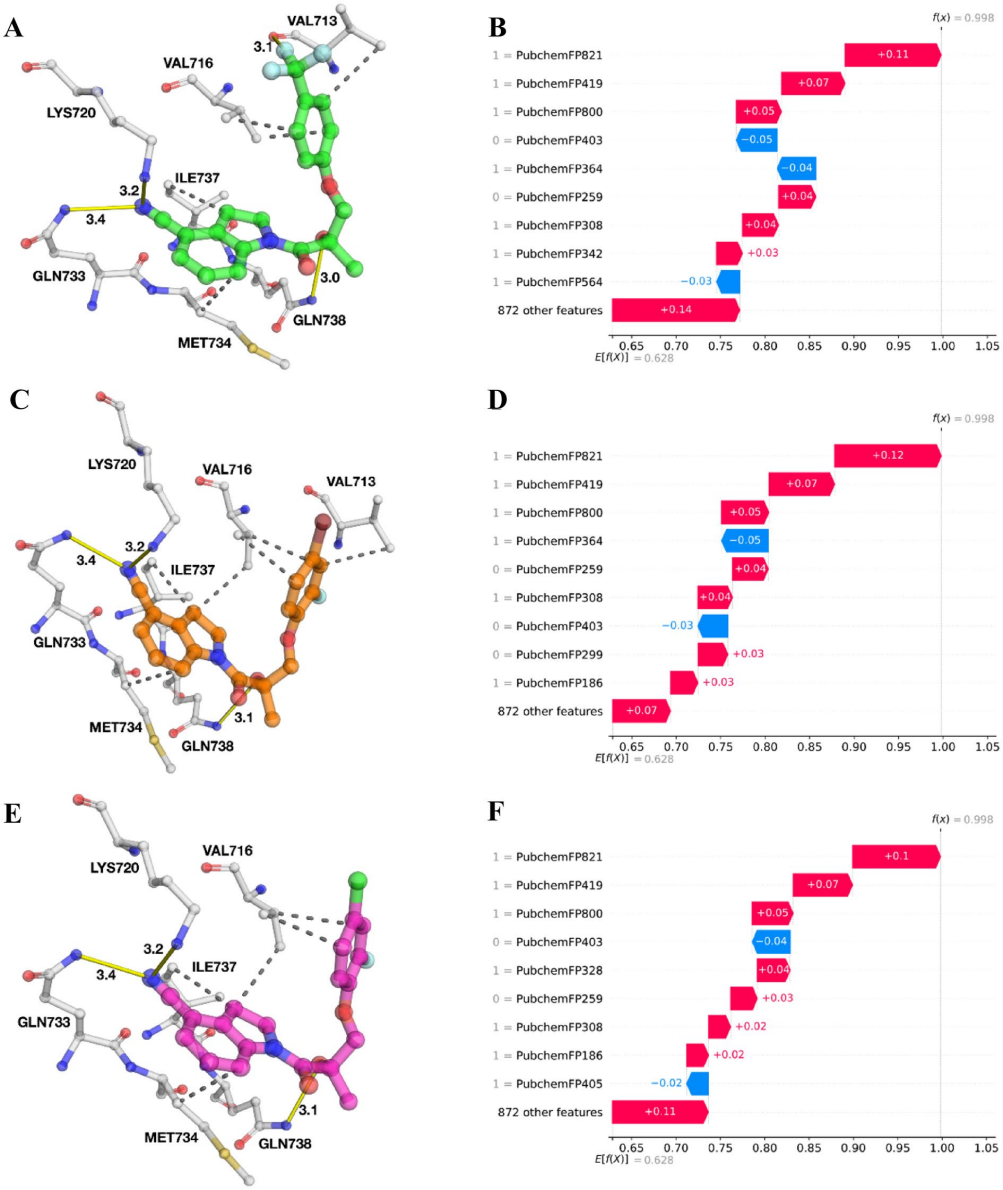
Close-up views of the binding interactions and corresponding SHAP waterfall plot of AR-CHEMBL3238281 (**A**) and (**B**), AR-CHEMBL3233070 (**C**) and (**D**) and AR-CHEMBL3233072 (**E**) and (**F**). Hydrogen bond and hydrophobic interactions are shown with straight line and dotted lines, respectively while SHAP values are shown in red (high value) and blue colours (low value) influencing the predictions toward positive and negative samples, respectively

Figure 5 shows the protein structure of AR with the top-three compounds (Additional file 1: Fig. S5) as determined by docking. The binding poses of the docked compounds in the AF2 binding site were flanked by residues of H3, H5, and H12. Upon binding of agonist or antagonist, H12 undergoes a conformational change which modulates AR activation. Structural analysis has revealed the role of key residues (i.e., Val716, Lys720, Met734, Ile737, Gln738, Met894, and Glu897) involved in the binding of coactivator proteins which shows differential binding when bound to antagonist as compared to agonist. The structural change of H12 is a key factor that blocks the AF2 site from binding to coactivator protein [15]. Figure 6 illustrates the residues involved in making polar and hydrophobic contacts between the AF2 allosteric site and the top three compounds. As can be observed from Fig. 6A, C, E, all three compounds form hydrogen bonds with Lys720, Gln733, and Gln738with the exception of CHEMBL3238281 which has an extra hydrogen bond with Val713. In addition, hydrophobic interactions were observed with residues Val713, Val716, Met734, and Ile737 for all the three compounds with the exception of CHEMBL3233070 which did not form a hydrophobic interaction with Val713. Interestingly, Val713 has not previously been observed as a residue involved in hydrophobic interactions to either co-activator or the antagonist ligand (co-crystal structure) of the AR protein. Hence, contact made with these residues by the top three compounds may contribute to the overall antagonistic effect.

Taking it a step further, we applied the combination of LGBM-PubChem and SHAP waterfall plots on the top-ten individual compounds to elucidate their features governing substructures for being AR antagonists (Fig. 6 and Additional file 1: Fig. S5). Figure 6B, D, F shows the SHAP waterfall plots of the top-three compounds. The contribution of each input value, either positive or negative, is highlighted through the SHAP waterfall plot towards the overall plausibility of a compound being an AR antagonist. As can be observed, the top-three features (i.e., PubChemFP821, PubChemFP419, and PubChemFP800) were present in all of the top-three compounds with high SHAP value (red colour) for all the compounds. Of note, the top-three features present in all compounds were also shown to be the top-three features in the top-twenty informative features mentioned above (Fig. 3). This indicates that PubChemFP821, PubChemFP419, and PubChemFP800 could be important substructure features for being AR antagonists and they pertain to 2-methylcyclohexan-1-amine, cyano group and 3-methylcyclohexan-1-amine (Table 5), respectively which have been discussed in the previous section.

Delving deeper into the individual compound features to investigate the underlying scaffold structures, it can be observed that all three compounds have an indoline-4-carbonitrile backbone and differ only at their side chains (Additional file 1: Fig. S4). The indole ring moiety forming part of the *N*-heterocyclics are commonly found in the natural environment and have been utilized as structural components of many therapeutic drugs for the treatment of microbial infections, cancers and inflammation [65]. Besides the top three features, PubChem342, PubChem299, and PubChem328 are unique contributing features for CHEMBL3238281, CHEMBL3233070, and CHEMBL3233072 respectively. Along with these, PubChem259 and PubChem308 corresponding to cyclic rings and hydroxide (OH) group, respectively were significant contributing features present in the compounds. PubChem342 pertains to fluromethane which is directly correlated to the trifluromethyl group (CF_3_) seen in CHEMBL3238281. The primary feature contributing trifluoromethyl analogue allows for the formation of hydrogen bond with Val713 in the AF2 site which is absent in the interactions of the other two compounds (Fig. 6A, C, E). 20–25% of pharmaceutical drugs contain fluorine either by direct fluorination or by incorporation of fluorinated functional groups. The existence of fluorine in these drugs has been shown to influence hydrogen bonding and electrostatic interactions of bound ligands [66, 67]. Furthermore, non-steroidal selective androgen receptor modulators (SARMs) such as Enobosarm, contains a CF3 group and has been fast-tracked by the FDA for the treatment of patients with AR-positive, ER-positive, and human epidermal growth factor receptor 2 (HER2)-negative metastatic breast cancer, based on data from the phase 3 ARTEST clinical trial [68]. In addition, the influence of trifluoromethyl can be due to their strong electron-withdrawing property [69–71].

PubChem299 feature corresponds to N–H which is part of the pyrrole heterocyclic ring forming the indole substructure of CHEMBL3233070 (Additional file 1: Fig. S4). Interestingly, the pyrrole moiety is present in various active compounds exhibiting anticancer, antibacterial, anti-inflammatory and anti-hypertensive properties [72]. Numerous research into the potential of pyrrole and its derivatives as a highly active scaffold has previously been explored [73–75]. In addition, recent studies pertaining to pyrrole-imidazole modified compounds have shown potency against castration resistant prostate cancers which develop through resistance to androgen depletion therapy [76] and enzalutamide-resistant prostate cancers activated by an alternative nuclear hormone receptor such as GR [77]. Thus, compounds containing this privileged substructure are promising for future investigations.

PubChem328 corresponds to isopropyl bromide which is a halogenated hydrocarbon. CHEMBL3233072 has a bromine substituent as part of its molecule. Although the Br substituent does not make direct interactions with residues in the AF2 binding pocket (Fig. 6E), it could still contribute through atomic parameter contributions (i.e., electrostatic or Van der Waals interactions). In addition, the presence of halogen (Cl, F, and Br)-substituted compounds were shown to have remarkable inhibitory activity when compared with electron-donating substituents as deduced from SAR studies [78, 79]. Intriguingly, several research has indicated the potency of brominated small molecule derivatives which have displayed anti-cancer activity toward both prostate and breast cancer cells while exhibiting no viable effect on noncancer cells [80–82]. Therefore, halogenated compounds warrant further investigation for their role as potential AR inhibitors. Taken together, the feature importance analysis based on SHAP and their contributions towards candidate AR antagonists predicted by our proposed framework, provide useful insights into further design and development of AR antagonists.

### DeepAR webserver

Herein, a webserver for our proposed model DeepAR, has been constructed to provide the scientific community with a practical tool that can be widely used for performing high-throughput identification of AR antagonists in an economic manner. Precisely, the chemical compound of interest is input as a SMILES notation into the DeepAR webserver after which, the prediction results are attained. A step-by-step guideline on the usage of the webserver is available for access at http://pmlabstack.pythonanywhere.com/about_DeepAR. This user-friendly web server is available at http://pmlabstack.pythonanywhere.com/DeepAR.

## Conclusion

In this study, we have presented DeepAR, which is a DL-based hybrid framework for accurate AR antagonist identification in an economic manner. Specifically, DeepAR was constructed by using a collection of 156 baseline models trained with 12 types of molecular descriptors and 13 different ML algorithms. Then, all the 156 baseline models were used to generate 156 PFs. Finally, the combination of all the 156 PFs were inputted into 1D-CNN for the construction of the meta-model by using the stacking strategy. The major contributions of DeepAR are as follows: (i) DeepAR is the first stacked ensemble learning framework designed for the identification and interpretation of AR antagonists. Remarkably, DeepAR is able to identify AR antagonists by using the SMILES notation without the use of structural information, highlighting its great capability for the high-throughput identification of AR antagonists.; (ii) DeepAR is capable of extracting and learning the key information embedded in AR antagonists by integrating a total of 156 baseline models; (iii) Comparative analysis in terms of the independent test dataset was sufficient to demonstrate the superior performance of DeepAR compared with several conventional ML classifiers, by achieving ACC of 0.911, MCC of 0.823, and AUC of 0.945; (iv) The SHAP-derived important features can determine the contributions of individual components for being AR antagonists which attribute to *N*-heterocyclics, halogenated substituents and cyano group; (v) Molecular docking highlights the interactions of potential AR antagonists identified through DeepAR; and (vi) We implemented an online web server (at http://pmlabstack.pythonanywhere.com/DeepAR) to facilitate experimental researchers for the large-scale identification of novel AR antagonists for follow-up experimental validation.

## Supplementary Information

**Additional file 1: Table 1.** Hyperparameter search details for 13 different ML classifiers. **Table S2.** Cross-validation results of 156 single feature-based models developed using 13 different ML algorithms and 12 molecular descriptors. **Table S3.** Independent test results of 156 single feature-based models developed using 13 different ML algorithms and 12 molecular descriptors. **Table S4.** Average cross-validation results of each molecular descriptor over 13 different ML algorithms. **Table S5.** Cross-validation results of 13 different ML algorithms trained with the combination of the 12 molecular descriptors. **Table S6.** Independent test results of 13 different ML algorithms trained with the combination of the 12 molecular descriptors. **Figure S1.** Plot of molecular weight (MW) vs Ghose-Crippen-Viswanadhan octanol-water partition coefficient (ALogP) for compounds in the curated dataset. The plot allows simple visualization of the chemical space of inhibitors against AR, where active and inactive compounds are shown in peach and teal colors, respectively. **Figure S2.** Box plots of Lipinski’s rule-of-five descriptors. The four rule-of-five descriptors are shown where (**A**) molecular weight (MW), (**B**) Ghose-Crippen-Viswanadhan octanol-water partition coefficient (ALogP), (**C**) hydrogen bond donor (nHBDon) and (**D**) hydrogen bond acceptor (nHBAcc), where active and inactive compounds are depicted in peach and teal colors, respectively. **Figure S3.** Box plots of molecular complexity descriptors. The four descriptors shown in this figure represent (**A**) aromatic ratio (ARR), (**B**) number of rings (nCIC), (**C**) number of rotatable bonds (RBN) and (**D**) number of benzene-like rings (nBnz), where active and inactive compounds are depicted in peach and teal colors, respectively. **Figure S5.** SHAP waterfall plots of the top ten-ranked compounds. CHEMBL3238279 (**A**), CHEMBL3233069 (**B**), CHEMBL3238280 (**C**), CHEMBL3238276 (**D**), CHEMBL3233070 (**E**), CHEMBL3238274 (F), CHEMBL3238278 (**G**), CHEMBL3238277 (**H**), CHEMBL3238281 (**I**), and CHEMBL3233072 (**J**), respectively.

## Data Availability

All the data used in this study are available at http://pmlabstack.pythonanywhere.com/DeepAR. Meanwhile, the source code is available at https://github.com/plenoi/DeepAR.
