# Supplementary material for: DeepAR: a novel deep learning-based hybrid framework for the interpretable prediction of androgen receptor antagonists
Source: J Cheminform. 2023 May 6;15:50. doi: 10.1186/s13321-023-00721-z (PMC10163717; doi:10.1186/s13321-023-00721-z)
Supplement: Supplementary file 1 — Additional file 1: Table 1. Hyperparameter search details for 13 different ML classifiers. Table S2. Cross-validation results of 156 single feature-based models developed using 13 different ML algorithms and 12 molecular descriptors. Table S3. Independent test results of 156 single feature-based models developed using 13 different ML algorithms and 12 molecular descriptors. Table S4. Average cross-validation results of each molecular descriptor over 13 different ML algorithms. Table S5. Cross-validation results of 13 different ML algorithms trained with the combination of the 12 molecular descriptors. Table S6. Independent test results of 13 different ML algorithms trained with the combination of the 12 molecular descriptors. Figure S1. Plot of molecular weight (MW) vs Ghose-Crippen-Viswanadhan octanol-water partition coefficient (ALogP) for compounds in the curated dataset. The plot allows simple visualization of the chemical space of inhibitors against AR, where active and inactive compounds are shown in peach and teal colors, respectively. Figure S2. Box plots of Lipinski’s rule-of-five descriptors. The four rule-of-five descriptors are shown where (A) molecular weight (MW), (B) Ghose-Crippen-Viswanadhan octanol-water partition coefficient (ALogP), (C) hydrogen bond donor (nHBDon) and (D) hydrogen bond acceptor (nHBAcc), where active and inactive compounds are depicted in peach and teal colors, respectively. Figure S3. Box plots of molecular complexity descriptors. The four descriptors shown in this figure represent (A) aromatic ratio (ARR), (B) number of rings (nCIC), (C) number of rotatable bonds (RBN) and (D) number of benzene-like rings (nBnz), where active and inactive compounds are depicted in peach and teal colors, respectively. Figure S5. SHAP waterfall plots of the top ten-ranked compounds. CHEMBL3238279 (A), CHEMBL3233069 (B), CHEMBL3238280 (C), CHEMBL3238276 (D), CHEMBL3233070 (E), CHEMBL3238274 (F), CHEMBL3238278 (G), CHEMBL3238277 (H), CHEMBL3238281 [file 13321_2023_721_MOESM1_ESM.docx]

# **DeepAR: a novel deep learning-based hybrid framework for the interpretable prediction of androgen receptor antagonists**

Nalini Schaduangrat^1^, Nuttapat Anuwongcharoen^2^, Phasit Charoenkwan^3,*^, Watshara Shoombuatong^1,*^

^1^ Center for Research Innovation and Biomedical Informatics, Faculty of Medical Technology, Mahidol University, Bangkok 10700, Thailand.

^2^ Department of Community Medical Technology, Faculty of Medical Technology, Mahidol University, Bangkok 10700, Thailand.

^3^ Modern Management and Information Technology, College of Arts, Media and Technology, Chiang Mai University, Chiang Mai 50200, Thailand.

^*^Correspondence should be addressed to P. Charoenkwan ([phasit.c@cmu.ac.th](mailto:phasit.c@cmu.ac.th)) and W. Shoombuatong; ([watshara.sho@mahidol.ac.th](mailto:watshara.sho@mahidol.ac.th)).

# **Supplementary Figure**

**Figure S1** Plot of molecular weight (MW) vs Ghose-Crippen-Viswanadhan octanol-water partition coefficient (ALogP) for compounds in the curated dataset. The plot allows simple visualization of the chemical space of inhibitors against AR, where active and inactive compounds are shown in peach and teal colors, respectively.

**
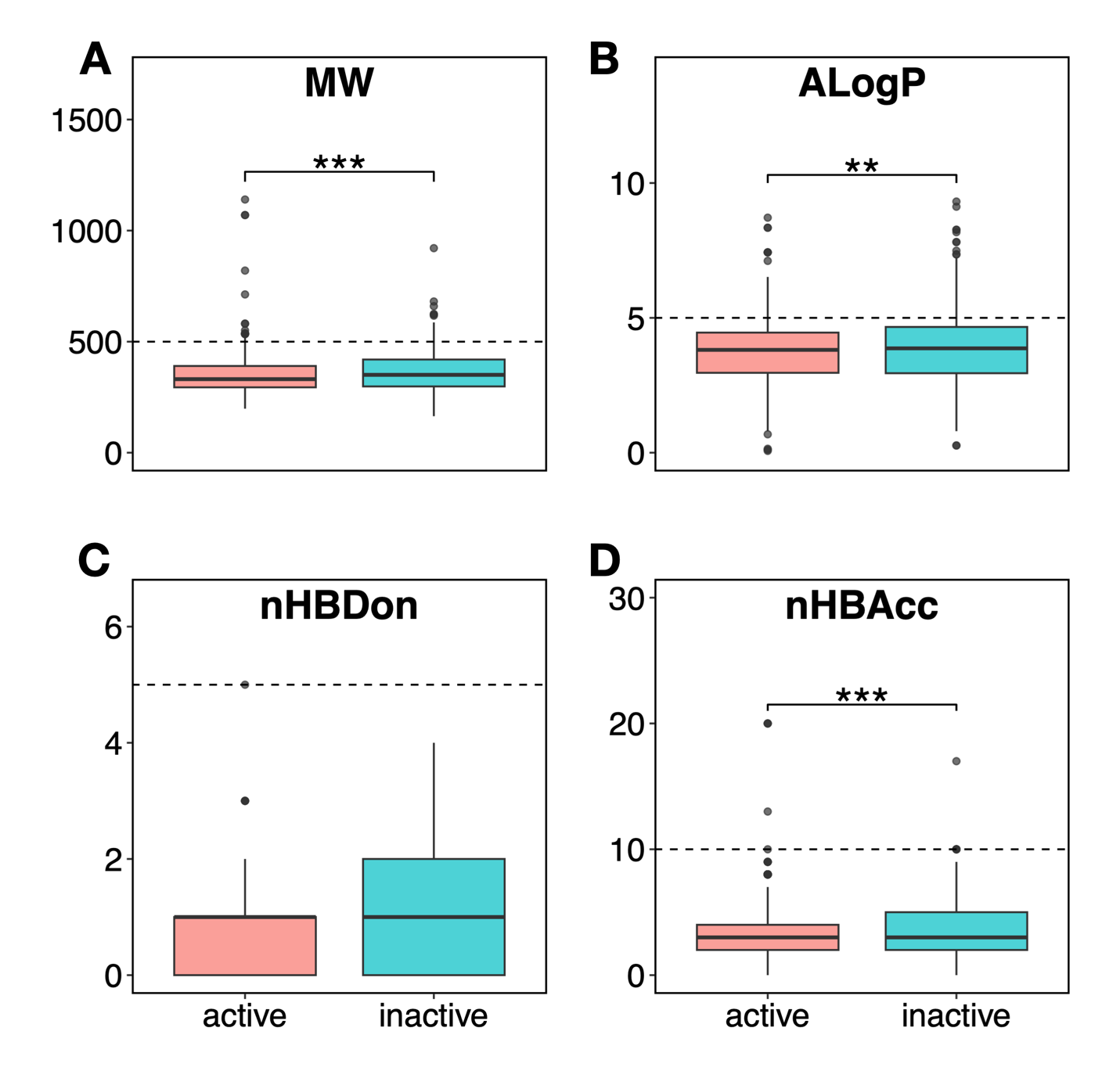
**

**Figure S2** Box plots of Lipinski’s rule-of-five descriptors. The four rule-of-five descriptors are shown where (**A**) molecular weight (MW), (**B**) Ghose-Crippen-Viswanadhan octanol-water partition coefficient (ALogP), (**C**) hydrogen bond donor (nHBDon) and (**D**) hydrogen bond acceptor (nHBAcc), where active and inactive compounds are depicted in peach and teal colors, respectively.

**
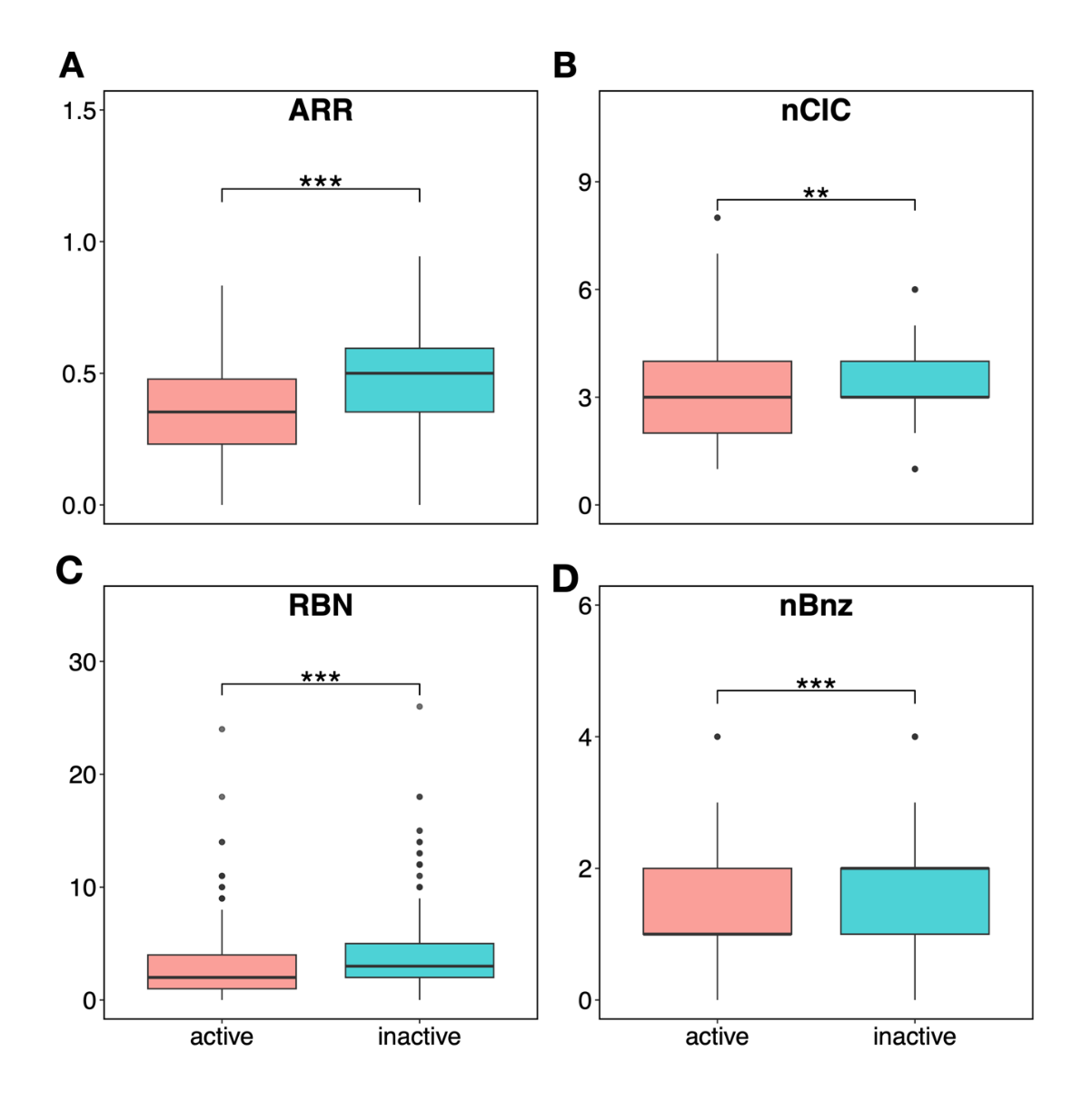
**

**Figure S3** Box plots of molecular complexity descriptors. The four descriptors shown in this figure represent (**A**) aromatic ratio (ARR), (**B**) number of rings (nCIC), (**C**) number of rotatable bonds (RBN) and (**D**) number of benzene-like rings (nBnz), where active and inactive compounds are depicted in peach and teal colors, respectively.

**
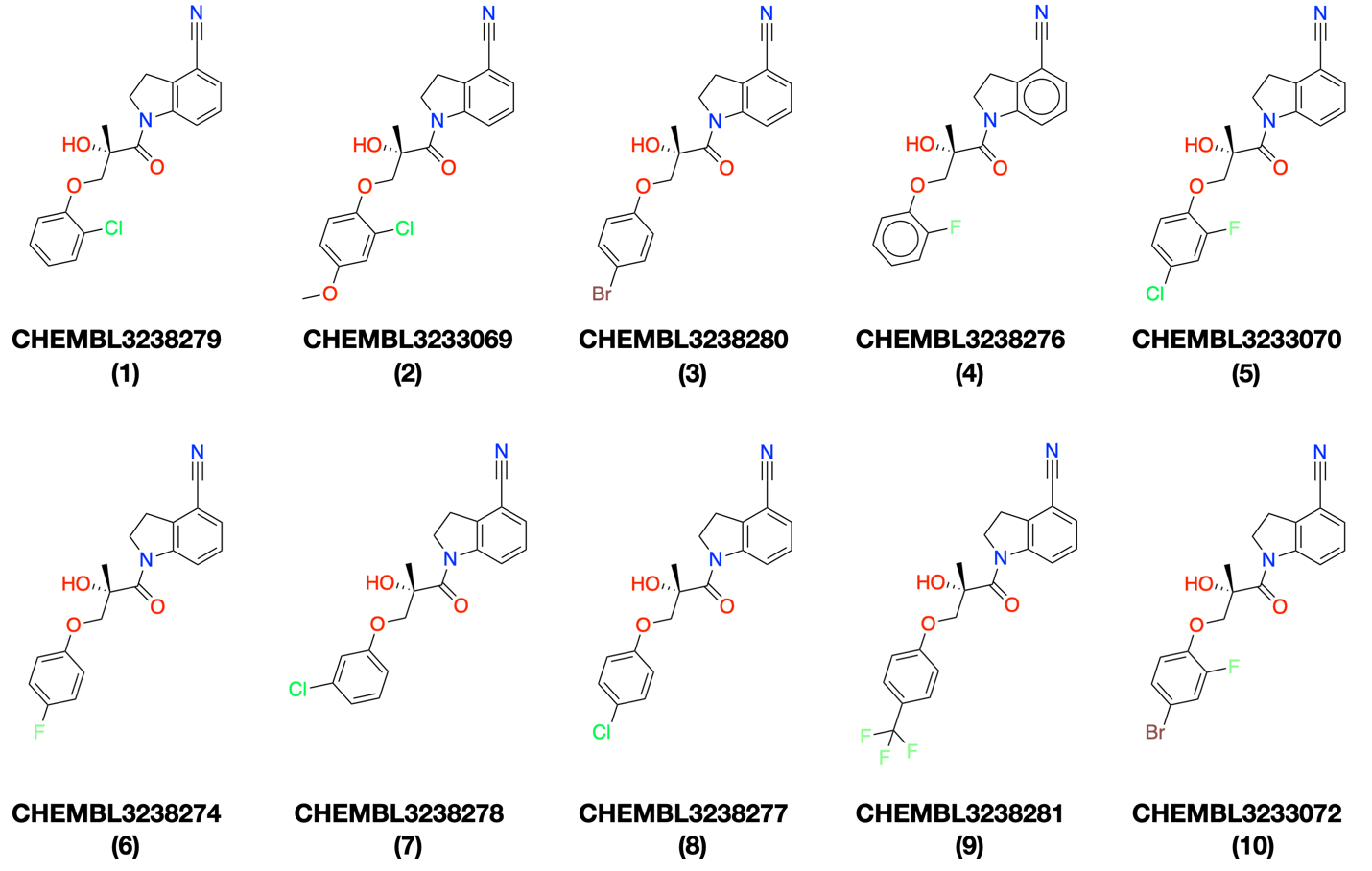
**

**Figure S4** Top-ten compounds ranked by their probability scores derived from DeepAR. The compounds are represented by numbers and their corresponding IUPAC names are in text. The set of top-ten compounds includes (S)-1-(3-(2-chlorophenoxy)-2-hydroxy-2-methylpropanoyl)indoline-4-carbonitrile **(1)**, (S)-1-(3-(2-chloro-4-methoxyphenoxy)-2-hydroxy-2-methylpropanoyl)indoline-4-carbonitrile **(2)**, (S)-1-(3-(4-bromophenoxy)-2-hydroxy-2-methylpropanoyl)indoline-4-carbonitrile **(3)**, (S)-1-(3-(2-fluorophenoxy)-2-hydroxy-2-methylpropanoyl)indoline-4-carbonitrile **(4)**, (S)-1-(3-(4-chloro-2-fluorophenoxy)-2-hydroxy-2-methylpropanoyl)indoline-4-carbonitrile **(5)**, (S)-1-(3-(4-fluorophenoxy)-2-hydroxy-2-methylpropanoyl)indoline-4-carbonitrile **(6)**, (S)-1-(3-(3-chlorophenoxy)-2-hydroxy-2-methylpropanoyl)indoline-4-carbonitrile **(7)**, (S)-1-(3-(4-chlorophenoxy)-2-hydroxy-2-methylpropanoyl)indoline-4-carbonitrile **(8)**, (S)-1-(2-hydroxy-2-methyl-3-(4-(trifluoromethyl)phenoxy)propanoyl)indoline-4-carbonitrile **(9)**, and (S)-1-(3-(4-bromo-2-fluorophenoxy)-2-hydroxy-2-methylpropanoyl)indoline-4-carbonitrile **(10)**.


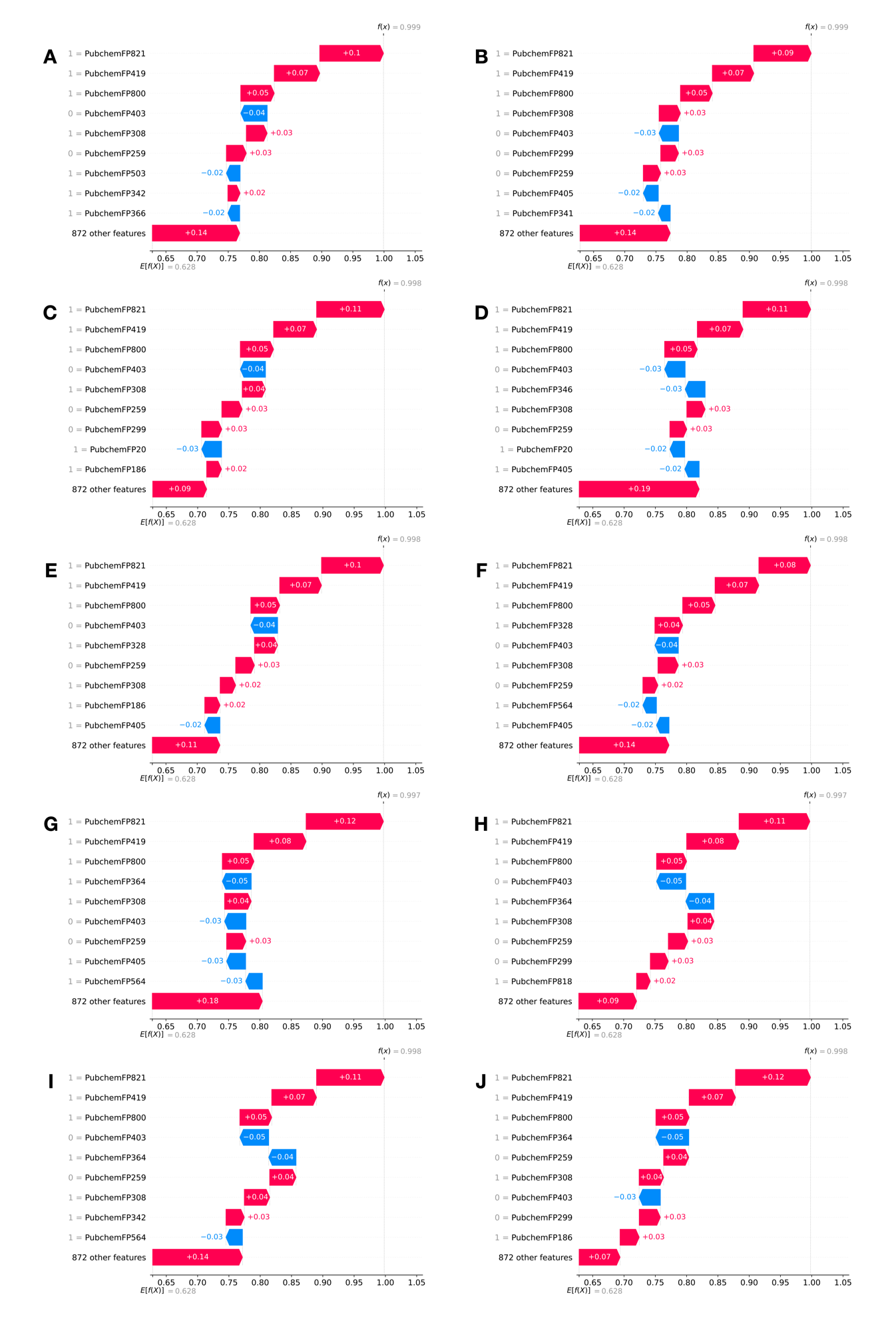


**Figure S5** SHAP waterfall plots of the top ten-ranked compounds. CHEMBL3238279 (A), CHEMBL3233069 (B), CHEMBL3238280 (C), CHEMBL3238276 (D), CHEMBL3233070 (E), CHEMBL3238274 (F), CHEMBL3238278 (G), CHEMBL3238277 (H), CHEMBL3238281 (I), and CHEMBL3233072 (J), respectively.

# **Additional Table**

**Table 1.** Hyperparameter search details for 13 different ML classifiers.

| **Method** | **Parameters** | **Range of parameters** |
| --- | --- | --- |
| ADA | n_estimators | [20, 50, 100, 200, 500] |
| DT | max_depth | Default |
| ET | n_estimators | [20, 50, 100, 200, 500] |
| KNN | number of neighbours | Default |
| LGBM | n_estimators | [20, 50, 100, 200, 500] |
| LR | C | [0.001, 0.01, 0.1, 1, 10, 100] |
| MLP | hidden_layer_sizes | [50, 100, 300, 500] |
| NB | standard_deviation() | Default |
| PLS | #Components | Default |
| RF | n_estimators | [20, 50, 100, 200, 500] |
| SVMLN | Cost | [2^0^–2^5^] in log_2_ steps |
| SVMRBF | Cost | [2^-4^–2^4^] in log_2_ steps |
| XGB | n_estimators | [20, 50, 100, 200, 500] |

Columns 2 and 3 represents the parameter name used in the Scikit-learn library and the range of parameter used to develop the model, respectively.

**Table S2** Cross-validation results of 156 single feature-based models developed using 13 different ML algorithms and 12 molecular descriptors.

| **Feature** | **Method** | **ACC** | **Sn** | **Sp** | **MCC** | **AUC** | **F1** | **Parameter** |
| --- | --- | --- | --- | --- | --- | --- | --- | --- |
| AP2D | ADA | 0.773 | 0.777 | 0.768 | 0.547 | 0.845 | 0.778 | 100 |
|  | DT | 0.789 | 0.823 | 0.752 | 0.580 | 0.800 | 0.799 | NA |
|  | ET | 0.817 | 0.829 | 0.804 | 0.636 | 0.892 | 0.823 | 20 |
|  | KNN | 0.814 | 0.867 | 0.758 | 0.632 | 0.813 | 0.827 | NA |
|  | LGBM | 0.837 | 0.846 | 0.826 | 0.675 | 0.902 | 0.841 | 100 |
|  | LR | 0.796 | 0.800 | 0.792 | 0.595 | 0.872 | 0.802 | 0.1 |
|  | MLP | 0.820 | 0.844 | 0.795 | 0.644 | 0.883 | 0.829 | 200 |
|  | NB | 0.593 | 0.960 | 0.205 | 0.249 | 0.744 | 0.708 | NA |
|  | PLS | 0.722 | 0.766 | 0.676 | 0.446 | 0.808 | 0.740 | NA |
|  | RF | 0.834 | 0.832 | 0.834 | 0.670 | 0.907 | 0.836 | 100 |
|  | SVMLN | 0.811 | 0.832 | 0.789 | 0.626 | 0.863 | 0.819 | 1 |
|  | SVMRBF | 0.825 | 0.843 | 0.804 | 0.652 | 0.883 | 0.832 | 16 |
|  | XGB | 0.838 | 0.861 | 0.813 | 0.678 | 0.907 | 0.845 | 200 |
| Circle | ADA | 0.828 | 0.823 | 0.832 | 0.656 | 0.871 | 0.830 | 200 |
|  | DT | 0.783 | 0.789 | 0.777 | 0.571 | 0.783 | 0.788 | NA |
|  | ET | 0.877 | 0.901 | 0.850 | 0.755 | 0.933 | 0.883 | 20 |
|  | KNN | 0.865 | 0.896 | 0.831 | 0.733 | 0.864 | 0.872 | NA |
|  | LGBM | 0.878 | 0.890 | 0.865 | 0.758 | 0.938 | 0.882 | 200 |
|  | LR | 0.860 | 0.878 | 0.841 | 0.721 | 0.930 | 0.866 | 0.1 |
|  | MLP | 0.860 | 0.890 | 0.829 | 0.723 | 0.919 | 0.867 | 100 |
|  | NB | 0.789 | 0.769 | 0.810 | 0.579 | 0.808 | 0.789 | NA |
|  | PLS | 0.853 | 0.872 | 0.832 | 0.707 | 0.928 | 0.859 | NA |
|  | RF | 0.871 | 0.896 | 0.844 | 0.743 | 0.940 | 0.877 | 200 |
|  | SVMLN | 0.835 | 0.861 | 0.807 | 0.673 | 0.897 | 0.842 | 1 |
|  | SVMRBF | 0.874 | 0.887 | 0.859 | 0.748 | 0.942 | 0.878 | 2 |
|  | XGB | 0.854 | 0.870 | 0.838 | 0.710 | 0.919 | 0.860 | 100 |
| CKD | ADA | 0.837 | 0.835 | 0.838 | 0.677 | 0.878 | 0.841 | 100 |
|  | DT | 0.819 | 0.846 | 0.789 | 0.639 | 0.819 | 0.827 | NA |
|  | ET | 0.860 | 0.878 | 0.841 | 0.722 | 0.923 | 0.867 | 200 |
|  | KNN | 0.844 | 0.884 | 0.801 | 0.690 | 0.843 | 0.854 | NA |
|  | LGBM | 0.863 | 0.878 | 0.847 | 0.728 | 0.926 | 0.869 | 200 |
|  | LR | 0.856 | 0.875 | 0.836 | 0.715 | 0.927 | 0.863 | 0.1 |
|  | MLP | 0.859 | 0.878 | 0.838 | 0.722 | 0.918 | 0.865 | 200 |
|  | NB | 0.750 | 0.884 | 0.609 | 0.516 | 0.855 | 0.785 | NA |
|  | PLS | 0.837 | 0.858 | 0.814 | 0.675 | 0.905 | 0.844 | NA |
|  | RF | 0.860 | 0.878 | 0.841 | 0.722 | 0.928 | 0.867 | 100 |
|  | SVMLN | 0.826 | 0.849 | 0.802 | 0.653 | 0.879 | 0.834 | 1 |
|  | SVMRBF | 0.868 | 0.875 | 0.860 | 0.738 | 0.929 | 0.872 | 1 |
|  | XGB | 0.847 | 0.869 | 0.823 | 0.697 | 0.922 | 0.854 | 200 |
| CKDExt | ADA | 0.840 | 0.872 | 0.805 | 0.681 | 0.880 | 0.848 | 100 |
|  | DT | 0.823 | 0.837 | 0.807 | 0.649 | 0.824 | 0.828 | NA |
|  | ET | 0.863 | 0.879 | 0.847 | 0.728 | 0.918 | 0.869 | 100 |
|  | KNN | 0.845 | 0.881 | 0.807 | 0.694 | 0.844 | 0.855 | NA |
|  | LGBM | 0.857 | 0.867 | 0.847 | 0.715 | 0.921 | 0.862 | 100 |
|  | LR | 0.860 | 0.881 | 0.838 | 0.722 | 0.923 | 0.866 | 0.1 |
|  | MLP | 0.851 | 0.876 | 0.826 | 0.706 | 0.909 | 0.859 | 20 |
|  | NB | 0.731 | 0.899 | 0.554 | 0.486 | 0.832 | 0.774 | NA |
|  | PLS | 0.832 | 0.849 | 0.814 | 0.665 | 0.904 | 0.839 | NA |
|  | RF | 0.862 | 0.875 | 0.847 | 0.726 | 0.930 | 0.868 | 200 |
|  | SVMLN | 0.819 | 0.841 | 0.795 | 0.638 | 0.875 | 0.827 | 1 |
|  | SVMRBF | 0.869 | 0.887 | 0.851 | 0.740 | 0.927 | 0.875 | 2 |
|  | XGB | 0.868 | 0.896 | 0.838 | 0.737 | 0.919 | 0.874 | 200 |
| CKDGraph | ADA | 0.814 | 0.838 | 0.789 | 0.630 | 0.844 | 0.822 | 100 |
|  | DT | 0.831 | 0.818 | 0.845 | 0.662 | 0.840 | 0.832 | NA |
|  | ET | 0.851 | 0.847 | 0.857 | 0.705 | 0.910 | 0.854 | 200 |
|  | KNN | 0.826 | 0.890 | 0.759 | 0.657 | 0.824 | 0.841 | NA |
|  | LGBM | 0.857 | 0.841 | 0.875 | 0.716 | 0.914 | 0.858 | 200 |
|  | LR | 0.826 | 0.838 | 0.814 | 0.654 | 0.878 | 0.831 | 1 |
|  | MLP | 0.828 | 0.826 | 0.829 | 0.657 | 0.890 | 0.831 | 200 |
|  | NB | 0.676 | 0.816 | 0.530 | 0.380 | 0.772 | 0.702 | NA |
|  | PLS | 0.750 | 0.812 | 0.685 | 0.504 | 0.835 | 0.769 | NA |
|  | RF | 0.859 | 0.841 | 0.878 | 0.720 | 0.920 | 0.859 | 200 |
|  | SVMLN | 0.814 | 0.826 | 0.801 | 0.628 | 0.869 | 0.820 | 1 |
|  | SVMRBF | 0.845 | 0.864 | 0.826 | 0.692 | 0.903 | 0.852 | 2 |
|  | XGB | 0.841 | 0.867 | 0.814 | 0.683 | 0.900 | 0.849 | 100 |
| Estate | ADA | 0.740 | 0.754 | 0.725 | 0.482 | 0.834 | 0.749 | 200 |
|  | DT | 0.795 | 0.827 | 0.762 | 0.592 | 0.813 | 0.806 | NA |
|  | ET | 0.819 | 0.841 | 0.795 | 0.638 | 0.904 | 0.827 | 200 |
|  | KNN | 0.820 | 0.928 | 0.706 | 0.653 | 0.817 | 0.842 | NA |
|  | LGBM | 0.819 | 0.838 | 0.798 | 0.638 | 0.895 | 0.826 | 200 |
|  | LR | 0.744 | 0.769 | 0.719 | 0.491 | 0.842 | 0.756 | 100 |
|  | MLP | 0.816 | 0.861 | 0.768 | 0.635 | 0.893 | 0.828 | 200 |
|  | NB | 0.581 | 0.959 | 0.181 | 0.220 | 0.742 | 0.702 | NA |
|  | PLS | 0.721 | 0.757 | 0.682 | 0.442 | 0.815 | 0.737 | NA |
|  | RF | 0.838 | 0.859 | 0.817 | 0.678 | 0.907 | 0.845 | 50 |
|  | SVMLN | 0.764 | 0.829 | 0.695 | 0.532 | 0.827 | 0.783 | 4 |
|  | SVMRBF | 0.827 | 0.832 | 0.823 | 0.656 | 0.890 | 0.832 | 2 |
|  | XGB | 0.811 | 0.821 | 0.801 | 0.624 | 0.889 | 0.817 | 200 |
| FP4 | ADA | 0.805 | 0.827 | 0.784 | 0.613 | 0.872 | 0.815 | 200 |
|  | DT | 0.814 | 0.858 | 0.768 | 0.632 | 0.819 | 0.826 | NA |
|  | ET | 0.869 | 0.910 | 0.825 | 0.742 | 0.908 | 0.878 | 50 |
|  | KNN | 0.838 | 0.928 | 0.743 | 0.687 | 0.835 | 0.855 | NA |
|  | LGBM | 0.854 | 0.875 | 0.832 | 0.711 | 0.914 | 0.861 | 100 |
|  | LR | 0.832 | 0.855 | 0.808 | 0.666 | 0.890 | 0.840 | 10 |
|  | MLP | 0.851 | 0.884 | 0.816 | 0.707 | 0.914 | 0.860 | 200 |
|  | NB | 0.716 | 0.811 | 0.615 | 0.463 | 0.807 | 0.733 | NA |
|  | PLS | 0.805 | 0.847 | 0.762 | 0.616 | 0.868 | 0.818 | NA |
|  | RF | 0.872 | 0.907 | 0.835 | 0.747 | 0.923 | 0.880 | 100 |
|  | SVMLN | 0.842 | 0.870 | 0.814 | 0.687 | 0.886 | 0.851 | 4 |
|  | SVMRBF | 0.860 | 0.890 | 0.829 | 0.722 | 0.916 | 0.867 | 4 |
|  | XGB | 0.853 | 0.884 | 0.820 | 0.708 | 0.914 | 0.861 | 200 |
| FP4C | ADA | 0.747 | 0.850 | 0.639 | 0.503 | 0.801 | 0.776 | 50 |
|  | DT | 0.727 | 0.887 | 0.557 | 0.474 | 0.796 | 0.770 | NA |
|  | ET | 0.741 | 0.881 | 0.594 | 0.500 | 0.827 | 0.778 | 50 |
|  | KNN | 0.695 | 0.971 | 0.404 | 0.456 | 0.688 | 0.767 | NA |
|  | LGBM | 0.700 | 0.645 | 0.759 | 0.408 | 0.777 | 0.688 | 100 |
|  | LR | 0.746 | 0.841 | 0.646 | 0.500 | 0.810 | 0.773 | 1 |
|  | MLP | 0.755 | 0.856 | 0.649 | 0.521 | 0.844 | 0.782 | 20 |
|  | NB | 0.669 | 0.900 | 0.427 | 0.398 | 0.804 | 0.727 | NA |
|  | PLS | 0.728 | 0.832 | 0.618 | 0.464 | 0.816 | 0.759 | NA |
|  | RF | 0.737 | 0.867 | 0.600 | 0.488 | 0.838 | 0.773 | 20 |
|  | SVMLN | 0.756 | 0.829 | 0.679 | 0.520 | 0.775 | 0.777 | 16 |
|  | SVMRBF | 0.759 | 0.838 | 0.676 | 0.526 | 0.818 | 0.782 | 1 |
|  | XGB | 0.732 | 0.844 | 0.615 | 0.475 | 0.810 | 0.764 | 200 |
| Hybrid | ADA | 0.857 | 0.881 | 0.832 | 0.717 | 0.896 | 0.864 | 100 |
|  | DT | 0.820 | 0.852 | 0.786 | 0.644 | 0.822 | 0.829 | NA |
|  | ET | 0.868 | 0.890 | 0.844 | 0.737 | 0.924 | 0.874 | 50 |
|  | KNN | 0.839 | 0.876 | 0.801 | 0.683 | 0.838 | 0.850 | NA |
|  | LGBM | 0.871 | 0.890 | 0.850 | 0.743 | 0.928 | 0.876 | 50 |
|  | LR | 0.871 | 0.881 | 0.860 | 0.742 | 0.924 | 0.875 | 1 |
|  | MLP | 0.872 | 0.890 | 0.853 | 0.749 | 0.916 | 0.878 | 200 |
|  | NB | 0.731 | 0.890 | 0.563 | 0.484 | 0.823 | 0.772 | NA |
|  | PLS | 0.823 | 0.829 | 0.817 | 0.647 | 0.908 | 0.828 | NA |
|  | RF | 0.874 | 0.881 | 0.866 | 0.748 | 0.936 | 0.878 | 200 |
|  | SVMLN | 0.860 | 0.870 | 0.850 | 0.722 | 0.905 | 0.864 | 1 |
|  | SVMRBF | 0.875 | 0.887 | 0.863 | 0.752 | 0.923 | 0.879 | 32 |
|  | XGB | 0.871 | 0.878 | 0.863 | 0.742 | 0.927 | 0.875 | 200 |
| KR | ADA | 0.844 | 0.884 | 0.802 | 0.691 | 0.891 | 0.853 | 100 |
|  | DT | 0.822 | 0.850 | 0.792 | 0.645 | 0.822 | 0.831 | NA |
|  | ET | 0.849 | 0.876 | 0.820 | 0.699 | 0.919 | 0.856 | 100 |
|  | KNN | 0.831 | 0.890 | 0.767 | 0.667 | 0.829 | 0.844 | NA |
|  | LGBM | 0.853 | 0.873 | 0.832 | 0.707 | 0.931 | 0.859 | 50 |
|  | LR | 0.847 | 0.876 | 0.817 | 0.695 | 0.928 | 0.855 | 1 |
|  | MLP | 0.853 | 0.905 | 0.798 | 0.709 | 0.927 | 0.864 | 50 |
|  | NB | 0.798 | 0.899 | 0.691 | 0.607 | 0.796 | 0.821 | NA |
|  | PLS | 0.823 | 0.876 | 0.768 | 0.651 | 0.909 | 0.836 | NA |
|  | RF | 0.857 | 0.896 | 0.816 | 0.717 | 0.930 | 0.866 | 100 |
|  | SVMLN | 0.825 | 0.858 | 0.789 | 0.651 | 0.901 | 0.834 | 1 |
|  | SVMRBF | 0.874 | 0.904 | 0.841 | 0.749 | 0.933 | 0.880 | 8 |
|  | XGB | 0.863 | 0.890 | 0.835 | 0.728 | 0.930 | 0.870 | 200 |
| MACCS | ADA | 0.826 | 0.841 | 0.811 | 0.653 | 0.891 | 0.833 | 200 |
|  | DT | 0.830 | 0.852 | 0.807 | 0.661 | 0.830 | 0.837 | NA |
|  | ET | 0.848 | 0.855 | 0.841 | 0.698 | 0.915 | 0.853 | 200 |
|  | KNN | 0.850 | 0.902 | 0.795 | 0.703 | 0.848 | 0.861 | NA |
|  | LGBM | 0.857 | 0.870 | 0.844 | 0.715 | 0.931 | 0.863 | 100 |
|  | LR | 0.831 | 0.844 | 0.817 | 0.662 | 0.897 | 0.837 | 1 |
|  | MLP | 0.854 | 0.867 | 0.841 | 0.709 | 0.924 | 0.860 | 200 |
|  | NB | 0.732 | 0.817 | 0.642 | 0.483 | 0.826 | 0.744 | NA |
|  | PLS | 0.798 | 0.800 | 0.796 | 0.597 | 0.875 | 0.802 | NA |
|  | RF | 0.854 | 0.864 | 0.844 | 0.712 | 0.925 | 0.859 | 50 |
|  | SVMLN | 0.828 | 0.835 | 0.820 | 0.656 | 0.887 | 0.832 | 1 |
|  | SVMRBF | 0.872 | 0.884 | 0.859 | 0.745 | 0.922 | 0.877 | 8 |
|  | XGB | 0.856 | 0.864 | 0.847 | 0.712 | 0.929 | 0.860 | 200 |
| PubChem | ADA | 0.835 | 0.847 | 0.823 | 0.673 | 0.886 | 0.841 | 200 |
|  | DT | 0.846 | 0.870 | 0.819 | 0.693 | 0.848 | 0.853 | NA |
|  | ET | 0.868 | 0.890 | 0.844 | 0.739 | 0.928 | 0.874 | 100 |
|  | KNN | 0.859 | 0.913 | 0.801 | 0.723 | 0.857 | 0.870 | NA |
|  | LGBM | 0.875 | 0.881 | 0.868 | 0.752 | 0.938 | 0.879 | 200 |
|  | LR | 0.850 | 0.866 | 0.832 | 0.701 | 0.919 | 0.855 | 1 |
|  | MLP | 0.865 | 0.884 | 0.844 | 0.733 | 0.927 | 0.871 | 200 |
|  | NB | 0.718 | 0.885 | 0.542 | 0.456 | 0.819 | 0.764 | NA |
|  | PLS | 0.829 | 0.875 | 0.780 | 0.663 | 0.889 | 0.841 | NA |
|  | RF | 0.863 | 0.893 | 0.832 | 0.729 | 0.931 | 0.871 | 20 |
|  | SVMLN | 0.837 | 0.858 | 0.814 | 0.674 | 0.904 | 0.844 | 1 |
|  | SVMRBF | 0.875 | 0.884 | 0.866 | 0.752 | 0.930 | 0.879 | 16 |
|  | XGB | 0.871 | 0.881 | 0.860 | 0.743 | 0.938 | 0.875 | 200 |

**Table S3** Independent test results of 156 single feature-based models developed using 13 different ML algorithms and 12 molecular descriptors.

| **Feature** | **Method** | **ACC** | **Sn** | **Sp** | **MCC** | **AUC** | **F1** | **Parameter** |
| --- | --- | --- | --- | --- | --- | --- | --- | --- |
| AP2D | ADA | 0.763 | 0.816 | 0.707 | 0.527 | 0.840 | 0.780 | 100 |
|  | DT | 0.822 | 0.920 | 0.720 | 0.655 | 0.820 | 0.842 | NA |
|  | ET | 0.840 | 0.851 | 0.829 | 0.680 | 0.910 | 0.846 | 20 |
|  | KNN | 0.846 | 0.897 | 0.793 | 0.694 | 0.845 | 0.857 | NA |
|  | LGBM | 0.858 | 0.874 | 0.841 | 0.716 | 0.915 | 0.864 | 100 |
|  | LR | 0.799 | 0.828 | 0.768 | 0.597 | 0.866 | 0.809 | 0.1 |
|  | MLP | 0.822 | 0.839 | 0.805 | 0.645 | 0.875 | 0.830 | 200 |
|  | NB | 0.615 | 0.977 | 0.232 | 0.316 | 0.770 | 0.723 | NA |
|  | PLS | 0.728 | 0.816 | 0.634 | 0.459 | 0.823 | 0.755 | NA |
|  | RF | 0.846 | 0.874 | 0.817 | 0.692 | 0.918 | 0.854 | 100 |
|  | SVMLN | 0.775 | 0.816 | 0.732 | 0.550 | 0.825 | 0.789 | 1 |
|  | SVMRBF | 0.817 | 0.862 | 0.768 | 0.634 | 0.894 | 0.829 | 16 |
|  | XGB | 0.834 | 0.885 | 0.780 | 0.670 | 0.914 | 0.846 | 200 |
| Circle | ADA | 0.817 | 0.851 | 0.780 | 0.633 | 0.875 | 0.827 | 200 |
|  | DT | 0.799 | 0.782 | 0.817 | 0.599 | 0.799 | 0.800 | NA |
|  | ET | 0.882 | 0.851 | 0.915 | 0.766 | 0.933 | 0.881 | 20 |
|  | KNN | 0.876 | 0.851 | 0.902 | 0.753 | 0.877 | 0.876 | NA |
|  | LGBM | 0.876 | 0.862 | 0.890 | 0.752 | 0.938 | 0.877 | 200 |
|  | LR | 0.876 | 0.839 | 0.915 | 0.754 | 0.944 | 0.874 | 0.1 |
|  | MLP | 0.888 | 0.874 | 0.902 | 0.776 | 0.944 | 0.889 | 100 |
|  | NB | 0.769 | 0.759 | 0.780 | 0.539 | 0.795 | 0.772 | NA |
|  | PLS | 0.840 | 0.816 | 0.866 | 0.682 | 0.916 | 0.840 | NA |
|  | RF | 0.888 | 0.851 | 0.927 | 0.778 | 0.941 | 0.886 | 200 |
|  | SVMLN | 0.870 | 0.851 | 0.890 | 0.741 | 0.938 | 0.871 | 1 |
|  | SVMRBF | 0.893 | 0.851 | 0.939 | 0.791 | 0.943 | 0.892 | 2 |
|  | XGB | 0.870 | 0.816 | 0.927 | 0.746 | 0.932 | 0.866 | 100 |
| CKD | ADA | 0.858 | 0.874 | 0.841 | 0.716 | 0.904 | 0.864 | 100 |
|  | DT | 0.870 | 0.885 | 0.854 | 0.739 | 0.875 | 0.875 | NA |
|  | ET | 0.882 | 0.874 | 0.890 | 0.763 | 0.928 | 0.884 | 200 |
|  | KNN | 0.876 | 0.908 | 0.841 | 0.752 | 0.875 | 0.883 | NA |
|  | LGBM | 0.882 | 0.885 | 0.878 | 0.763 | 0.943 | 0.885 | 200 |
|  | LR | 0.882 | 0.862 | 0.902 | 0.764 | 0.946 | 0.882 | 0.1 |
|  | MLP | 0.876 | 0.885 | 0.866 | 0.751 | 0.941 | 0.880 | 200 |
|  | NB | 0.728 | 0.862 | 0.585 | 0.467 | 0.814 | 0.765 | NA |
|  | PLS | 0.828 | 0.805 | 0.854 | 0.658 | 0.904 | 0.828 | NA |
|  | RF | 0.876 | 0.874 | 0.878 | 0.751 | 0.935 | 0.879 | 100 |
|  | SVMLN | 0.882 | 0.874 | 0.890 | 0.763 | 0.937 | 0.884 | 1 |
|  | SVMRBF | 0.858 | 0.828 | 0.890 | 0.718 | 0.937 | 0.857 | 1 |
|  | XGB | 0.882 | 0.874 | 0.890 | 0.763 | 0.943 | 0.884 | 200 |
| CKDExt | ADA | 0.864 | 0.851 | 0.878 | 0.728 | 0.930 | 0.865 | 100 |
|  | DT | 0.793 | 0.862 | 0.720 | 0.589 | 0.795 | 0.811 | NA |
|  | ET | 0.882 | 0.897 | 0.866 | 0.763 | 0.919 | 0.886 | 100 |
|  | KNN | 0.893 | 0.920 | 0.866 | 0.787 | 0.893 | 0.899 | NA |
|  | LGBM | 0.893 | 0.874 | 0.915 | 0.788 | 0.944 | 0.894 | 100 |
|  | LR | 0.870 | 0.839 | 0.902 | 0.742 | 0.940 | 0.869 | 0.1 |
|  | MLP | 0.870 | 0.874 | 0.866 | 0.739 | 0.942 | 0.874 | 20 |
|  | NB | 0.740 | 0.897 | 0.573 | 0.499 | 0.835 | 0.780 | NA |
|  | PLS | 0.781 | 0.782 | 0.780 | 0.562 | 0.892 | 0.786 | NA |
|  | RF | 0.876 | 0.874 | 0.878 | 0.751 | 0.938 | 0.879 | 200 |
|  | SVMLN | 0.852 | 0.862 | 0.841 | 0.704 | 0.925 | 0.857 | 1 |
|  | SVMRBF | 0.864 | 0.828 | 0.902 | 0.731 | 0.940 | 0.862 | 2 |
|  | XGB | 0.888 | 0.897 | 0.878 | 0.775 | 0.939 | 0.891 | 200 |
| CKDGraph | ADA | 0.817 | 0.782 | 0.854 | 0.636 | 0.865 | 0.814 | 100 |
|  | DT | 0.817 | 0.805 | 0.829 | 0.634 | 0.816 | 0.819 | NA |
|  | ET | 0.858 | 0.874 | 0.841 | 0.716 | 0.902 | 0.864 | 200 |
|  | KNN | 0.828 | 0.931 | 0.720 | 0.668 | 0.825 | 0.848 | NA |
|  | LGBM | 0.834 | 0.828 | 0.841 | 0.669 | 0.901 | 0.837 | 200 |
|  | LR | 0.828 | 0.816 | 0.841 | 0.657 | 0.877 | 0.830 | 1 |
|  | MLP | 0.834 | 0.828 | 0.841 | 0.669 | 0.884 | 0.837 | 200 |
|  | NB | 0.722 | 0.920 | 0.512 | 0.476 | 0.805 | 0.773 | NA |
|  | PLS | 0.722 | 0.759 | 0.683 | 0.443 | 0.814 | 0.737 | NA |
|  | RF | 0.828 | 0.839 | 0.817 | 0.656 | 0.903 | 0.834 | 200 |
|  | SVMLN | 0.834 | 0.828 | 0.841 | 0.669 | 0.871 | 0.837 | 1 |
|  | SVMRBF | 0.822 | 0.828 | 0.817 | 0.645 | 0.871 | 0.828 | 2 |
|  | XGB | 0.811 | 0.816 | 0.805 | 0.621 | 0.880 | 0.816 | 100 |
| Estate | ADA | 0.757 | 0.759 | 0.756 | 0.515 | 0.827 | 0.763 | 200 |
|  | DT | 0.799 | 0.782 | 0.817 | 0.599 | 0.812 | 0.800 | NA |
|  | ET | 0.864 | 0.828 | 0.902 | 0.731 | 0.909 | 0.862 | 200 |
|  | KNN | 0.870 | 0.908 | 0.829 | 0.741 | 0.869 | 0.878 | NA |
|  | LGBM | 0.858 | 0.816 | 0.902 | 0.720 | 0.928 | 0.855 | 200 |
|  | LR | 0.763 | 0.782 | 0.744 | 0.526 | 0.831 | 0.773 | 100 |
|  | MLP | 0.864 | 0.851 | 0.878 | 0.728 | 0.919 | 0.865 | 200 |
|  | NB | 0.633 | 0.989 | 0.256 | 0.363 | 0.810 | 0.735 | NA |
|  | PLS | 0.757 | 0.839 | 0.671 | 0.518 | 0.840 | 0.781 | NA |
|  | RF | 0.864 | 0.828 | 0.902 | 0.731 | 0.919 | 0.862 | 50 |
|  | SVMLN | 0.775 | 0.851 | 0.695 | 0.554 | 0.829 | 0.796 | 4 |
|  | SVMRBF | 0.781 | 0.736 | 0.829 | 0.566 | 0.873 | 0.776 | 2 |
|  | XGB | 0.805 | 0.770 | 0.841 | 0.612 | 0.882 | 0.802 | 200 |
| FP4 | ADA | 0.781 | 0.793 | 0.768 | 0.562 | 0.839 | 0.789 | 200 |
|  | DT | 0.870 | 0.885 | 0.854 | 0.739 | 0.883 | 0.875 | NA |
|  | ET | 0.911 | 0.908 | 0.915 | 0.822 | 0.919 | 0.913 | 50 |
|  | KNN | 0.882 | 0.908 | 0.854 | 0.764 | 0.881 | 0.888 | NA |
|  | LGBM | 0.870 | 0.828 | 0.915 | 0.743 | 0.947 | 0.867 | 100 |
|  | LR | 0.817 | 0.805 | 0.829 | 0.634 | 0.880 | 0.819 | 10 |
|  | MLP | 0.852 | 0.828 | 0.878 | 0.706 | 0.919 | 0.852 | 200 |
|  | NB | 0.716 | 0.851 | 0.573 | 0.443 | 0.751 | 0.755 | NA |
|  | PLS | 0.775 | 0.828 | 0.720 | 0.551 | 0.846 | 0.791 | NA |
|  | RF | 0.899 | 0.885 | 0.915 | 0.799 | 0.940 | 0.901 | 100 |
|  | SVMLN | 0.822 | 0.805 | 0.841 | 0.646 | 0.870 | 0.824 | 4 |
|  | SVMRBF | 0.858 | 0.828 | 0.890 | 0.718 | 0.927 | 0.857 | 4 |
|  | XGB | 0.858 | 0.851 | 0.866 | 0.716 | 0.930 | 0.860 | 200 |
| FP4C | ADA | 0.728 | 0.839 | 0.610 | 0.462 | 0.773 | 0.760 | 50 |
|  | DT | 0.716 | 0.874 | 0.549 | 0.448 | 0.778 | 0.760 | NA |
|  | ET | 0.728 | 0.885 | 0.561 | 0.474 | 0.808 | 0.770 | 50 |
|  | KNN | 0.686 | 0.931 | 0.427 | 0.417 | 0.679 | 0.753 | NA |
|  | LGBM | 0.663 | 0.655 | 0.671 | 0.326 | 0.759 | 0.667 | 100 |
|  | LR | 0.692 | 0.793 | 0.585 | 0.388 | 0.789 | 0.726 | 1 |
|  | MLP | 0.704 | 0.839 | 0.561 | 0.418 | 0.802 | 0.745 | 20 |
|  | NB | 0.675 | 0.943 | 0.390 | 0.402 | 0.749 | 0.749 | NA |
|  | PLS | 0.710 | 0.828 | 0.585 | 0.427 | 0.771 | 0.746 | NA |
|  | RF | 0.722 | 0.851 | 0.585 | 0.454 | 0.826 | 0.759 | 20 |
|  | SVMLN | 0.686 | 0.793 | 0.573 | 0.376 | 0.718 | 0.723 | 16 |
|  | SVMRBF | 0.704 | 0.805 | 0.598 | 0.412 | 0.795 | 0.737 | 1 |
|  | XGB | 0.704 | 0.816 | 0.585 | 0.414 | 0.803 | 0.740 | 200 |
| Hybrid | ADA | 0.834 | 0.816 | 0.854 | 0.670 | 0.898 | 0.835 | 100 |
|  | DT | 0.858 | 0.897 | 0.817 | 0.717 | 0.865 | 0.867 | NA |
|  | ET | 0.899 | 0.885 | 0.915 | 0.799 | 0.926 | 0.901 | 50 |
|  | KNN | 0.893 | 0.931 | 0.854 | 0.788 | 0.892 | 0.900 | NA |
|  | LGBM | 0.882 | 0.839 | 0.927 | 0.767 | 0.934 | 0.880 | 50 |
|  | LR | 0.864 | 0.851 | 0.878 | 0.728 | 0.940 | 0.865 | 1 |
|  | MLP | 0.864 | 0.862 | 0.866 | 0.728 | 0.936 | 0.867 | 200 |
|  | NB | 0.740 | 0.862 | 0.610 | 0.489 | 0.846 | 0.773 | NA |
|  | PLS | 0.852 | 0.828 | 0.878 | 0.706 | 0.899 | 0.852 | NA |
|  | RF | 0.917 | 0.920 | 0.915 | 0.834 | 0.935 | 0.920 | 200 |
|  | SVMLN | 0.852 | 0.874 | 0.829 | 0.704 | 0.925 | 0.859 | 1 |
|  | SVMRBF | 0.876 | 0.897 | 0.854 | 0.751 | 0.933 | 0.881 | 32 |
|  | XGB | 0.870 | 0.851 | 0.890 | 0.741 | 0.938 | 0.871 | 200 |
| KR | ADA | 0.828 | 0.816 | 0.841 | 0.657 | 0.882 | 0.830 | 100 |
|  | DT | 0.876 | 0.885 | 0.866 | 0.751 | 0.879 | 0.880 | NA |
|  | ET | 0.893 | 0.897 | 0.890 | 0.787 | 0.951 | 0.897 | 100 |
|  | KNN | 0.882 | 0.943 | 0.817 | 0.768 | 0.880 | 0.891 | NA |
|  | LGBM | 0.870 | 0.851 | 0.890 | 0.741 | 0.933 | 0.871 | 50 |
|  | LR | 0.882 | 0.885 | 0.878 | 0.763 | 0.940 | 0.885 | 1 |
|  | MLP | 0.870 | 0.862 | 0.878 | 0.740 | 0.927 | 0.872 | 50 |
|  | NB | 0.787 | 0.851 | 0.720 | 0.576 | 0.785 | 0.804 | NA |
|  | PLS | 0.828 | 0.862 | 0.793 | 0.657 | 0.897 | 0.838 | NA |
|  | RF | 0.870 | 0.897 | 0.841 | 0.740 | 0.949 | 0.876 | 100 |
|  | SVMLN | 0.852 | 0.828 | 0.878 | 0.706 | 0.938 | 0.852 | 1 |
|  | SVMRBF | 0.876 | 0.862 | 0.890 | 0.752 | 0.951 | 0.877 | 8 |
|  | XGB | 0.840 | 0.805 | 0.878 | 0.683 | 0.919 | 0.838 | 200 |
| MACCS | ADA | 0.834 | 0.816 | 0.854 | 0.670 | 0.920 | 0.835 | 200 |
|  | DT | 0.834 | 0.839 | 0.829 | 0.668 | 0.843 | 0.839 | NA |
|  | ET | 0.888 | 0.885 | 0.890 | 0.775 | 0.935 | 0.890 | 200 |
|  | KNN | 0.888 | 0.920 | 0.854 | 0.776 | 0.887 | 0.894 | NA |
|  | LGBM | 0.864 | 0.816 | 0.915 | 0.733 | 0.947 | 0.861 | 100 |
|  | LR | 0.858 | 0.862 | 0.854 | 0.716 | 0.922 | 0.862 | 1 |
|  | MLP | 0.870 | 0.851 | 0.890 | 0.741 | 0.949 | 0.871 | 200 |
|  | NB | 0.728 | 0.862 | 0.585 | 0.467 | 0.812 | 0.765 | NA |
|  | PLS | 0.751 | 0.736 | 0.768 | 0.504 | 0.844 | 0.753 | NA |
|  | RF | 0.882 | 0.874 | 0.890 | 0.763 | 0.934 | 0.884 | 50 |
|  | SVMLN | 0.834 | 0.828 | 0.841 | 0.669 | 0.925 | 0.837 | 1 |
|  | SVMRBF | 0.882 | 0.862 | 0.902 | 0.764 | 0.952 | 0.882 | 8 |
|  | XGB | 0.852 | 0.805 | 0.902 | 0.709 | 0.938 | 0.848 | 200 |
| PubChem | ADA | 0.870 | 0.851 | 0.890 | 0.741 | 0.941 | 0.871 | 200 |
|  | DT | 0.858 | 0.805 | 0.915 | 0.722 | 0.876 | 0.854 | NA |
|  | ET | 0.888 | 0.862 | 0.915 | 0.777 | 0.931 | 0.888 | 100 |
|  | KNN | 0.893 | 0.920 | 0.866 | 0.787 | 0.893 | 0.899 | NA |
|  | LGBM | 0.852 | 0.816 | 0.890 | 0.707 | 0.941 | 0.850 | 200 |
|  | LR | 0.882 | 0.862 | 0.902 | 0.764 | 0.938 | 0.882 | 1 |
|  | MLP | 0.888 | 0.897 | 0.878 | 0.775 | 0.938 | 0.891 | 200 |
|  | NB | 0.746 | 0.931 | 0.549 | 0.522 | 0.859 | 0.790 | NA |
|  | PLS | 0.775 | 0.805 | 0.744 | 0.550 | 0.874 | 0.787 | NA |
|  | RF | 0.870 | 0.839 | 0.902 | 0.742 | 0.929 | 0.869 | 20 |
|  | SVMLN | 0.888 | 0.874 | 0.902 | 0.776 | 0.935 | 0.889 | 1 |
|  | SVMRBF | 0.882 | 0.828 | 0.939 | 0.769 | 0.957 | 0.878 | 16 |
|  | XGB | 0.864 | 0.839 | 0.890 | 0.729 | 0.938 | 0.864 | 200 |

**Table S4** Average cross-validation results of each molecular descriptor over 13 different ML algorithms.

| **Feature** | **ACC** | **Sn** | **Sp** | **MCC** | **AUC** | **F1** |
| --- | --- | --- | --- | --- | --- | --- |
| AP2D | 0.790 | 0.837 | 0.740 | 0.587 | 0.855 | 0.806 |
| Circle | 0.848 | 0.863 | 0.832 | 0.698 | 0.898 | 0.853 |
| CKD | 0.840 | 0.868 | 0.811 | 0.684 | 0.896 | 0.849 |
| CKDExt | 0.840 | 0.872 | 0.806 | 0.684 | 0.893 | 0.850 |
| CKDGraph | 0.817 | 0.840 | 0.792 | 0.638 | 0.869 | 0.825 |
| Estate | 0.777 | 0.837 | 0.713 | 0.560 | 0.851 | 0.796 |
| FP4 | 0.832 | 0.873 | 0.789 | 0.669 | 0.882 | 0.842 |
| FP4C | 0.730 | 0.849 | 0.605 | 0.479 | 0.800 | 0.763 |
| Hybrid | 0.849 | 0.877 | 0.819 | 0.701 | 0.898 | 0.857 |
| KR | 0.841 | 0.883 | 0.798 | 0.686 | 0.896 | 0.851 |
| MACCS | 0.834 | 0.853 | 0.813 | 0.670 | 0.892 | 0.840 |
| PubChem | 0.845 | 0.879 | 0.810 | 0.695 | 0.901 | 0.855 |

**Table S5** Cross-validation results of 13 different ML algorithms trained with the combination of the 12 molecular descriptors.

| **Method** | **ACC** | **Sn** | **Sp** | **MCC** | **AUC** | **F1** |
| --- | --- | --- | --- | --- | --- | --- |
| ADA-All | 0.854 | 0.864 | 0.844 | 0.709 | 0.908 | 0.859 |
| DT-All | 0.826 | 0.844 | 0.808 | 0.653 | 0.826 | 0.833 |
| ET-All | 0.866 | 0.884 | 0.847 | 0.734 | 0.945 | 0.873 |
| KNN-All | 0.851 | 0.896 | 0.804 | 0.706 | 0.850 | 0.861 |
| LGBM-All | 0.880 | 0.887 | 0.871 | 0.760 | 0.936 | 0.883 |
| LR-All | 0.881 | 0.898 | 0.863 | 0.764 | 0.941 | 0.886 |
| MLP-All | 0.886 | 0.907 | 0.862 | 0.774 | 0.934 | 0.891 |
| NB-All | 0.802 | 0.815 | 0.789 | 0.606 | 0.803 | 0.810 |
| PLS-All | 0.850 | 0.861 | 0.838 | 0.701 | 0.927 | 0.854 |
| RF-All | 0.872 | 0.884 | 0.859 | 0.746 | 0.945 | 0.877 |
| SVMLN-All | 0.865 | 0.884 | 0.844 | 0.730 | 0.927 | 0.870 |
| SVMRBF-All | 0.883 | 0.898 | 0.866 | 0.766 | 0.942 | 0.888 |
| XGB-All | 0.881 | 0.901 | 0.859 | 0.764 | 0.937 | 0.886 |

**Table S6** Independent test results of 13 different ML algorithms trained with the combination of the 12 molecular descriptors.

| **Methods** | **ACC** | **Sn** | **Sp** | **MCC** | **AUC** | **F1** |
| --- | --- | --- | --- | --- | --- | --- |
| ADA-All | 0.882 | 0.885 | 0.878 | 0.763 | 0.910 | 0.885 |
| DT-All | 0.840 | 0.828 | 0.854 | 0.681 | 0.841 | 0.842 |
| ET-All | 0.888 | 0.874 | 0.902 | 0.776 | 0.941 | 0.889 |
| KNN-All | 0.882 | 0.897 | 0.866 | 0.763 | 0.881 | 0.886 |
| LGBM-All | 0.876 | 0.862 | 0.890 | 0.752 | 0.944 | 0.877 |
| LR-All | 0.888 | 0.862 | 0.915 | 0.777 | 0.949 | 0.888 |
| MLP-All | 0.888 | 0.874 | 0.902 | 0.776 | 0.949 | 0.889 |
| NB-All | 0.828 | 0.805 | 0.854 | 0.658 | 0.829 | 0.828 |
| PLS-All | 0.858 | 0.828 | 0.890 | 0.718 | 0.910 | 0.857 |
| RF-All | 0.893 | 0.874 | 0.915 | 0.788 | 0.943 | 0.894 |
| SVMLN-All | 0.882 | 0.885 | 0.878 | 0.763 | 0.945 | 0.885 |
| SVMRBF-All | 0.864 | 0.828 | 0.902 | 0.731 | 0.942 | 0.862 |
| XGB-All | 0.882 | 0.839 | 0.927 | 0.767 | 0.940 | 0.880 |
